# Usefulness of serum microRNA as a predictive marker of recurrence and prognosis in biliary tract cancer after radical surgery

**DOI:** 10.1038/s41598-019-42392-7

**Published:** 2019-04-11

**Authors:** Yu Akazawa, Shoichi Mizuno, Norihiro Fujinami, Toshihiro Suzuki, Yusuke Yoshioka, Takahiro Ochiya, Yasunari Nakamoto, Tetsuya Nakatsura

**Affiliations:** 1 Division of Cancer Immunotherapy, Exploratory Oncology Research and Clinical Trial Center, National Cancer Center, Kashiwa, Japan; 2 Second Department of Internal Medicine, Faculty of Medical Sciences, University of Fukui, Fukui, Japan; 3 Division of Molecular and Cellular Medicine, National Cancer Center Research Institute, Tokyo, Japan; 4 Institute of Medical Science, Tokyo Medical University, Tokyo, Japan

## Abstract

Biliary tract cancer (BTC) is an aggressive type of malignant tumour. Even after radical resection, the risk of recurrence is still high, resulting in a poor prognosis. Here, we investigated the usefulness of serum miRNAs as predictive markers of recurrence and prognosis for patients with BTC after radical surgery using 66 serum samples that were collected at three time points from 22 patients with BTC who underwent radical surgery. Using microarray analysis, we successfully identified six specific miRNAs (*miR-1225-3p*, *miR-1234-3p*, *miR1260b*, *miR-1470*, *miR-6834-3p*, and *miR-6875-5p*) associated with recurrence and prognosis of BTC after radical surgery. In addition, using a combination of these miRNAs, we developed a recurrence predictive index to predict recurrence in patients with BTC after operation with high accuracy. Patients having higher index scores (≥ cut-off) had significantly worse recurrence-free survival (RFS) and overall survival (OS) than those with lower index scores (<cut-off). Furthermore, the index was an independent factor related to RFS and OS by univariate and multivariate analyses using a Cox hazard proportional model. Overall, our results provided compelling evidence for the potential usefulness of specific serum miRNAs as effective predictive tools for recurrence and prognosis in patients with BTC who underwent radical surgery.

## Introduction

Biliary tract cancer (BTC) is as one of the most aggressive types of malignant tumours and is associated with a poor prognosis. In Japan, approximately 18,000 cases of BTC were diagnosed from 2008 to 2013^1^. In the United States of America in 2017, approximately 12,000 new cases of BTC were diagnosed, and over 3,800 BTC-related deaths occurred^2^. Indeed, in recent years, the incidence of BTC has increased worldwide. Although ultrasonography, computed tomography, and magnetic resonance imaging are usually used for the diagnosis of BTC, these imaging modalities are inadequate for the early diagnosis of BTC due to its growth along the bile duct. In addition, patients with BTC are typically asymptomatic at early stages of the disease, and BTC is often diagnosed at an advanced stage, resulting in a poor prognosis; the 5-year survival rate in these patients is less than 5%^3^. The most promising treatment for BTC is limited to surgical resection, and early diagnosis of BTC at a resectable stage is crucial for improving survival rates in patients with BTC. However, even if radical resection has been performed at a resectable stage, the risks of recurrence and metastasis are still serious^4,5^.

Carbohydrate antigen (CA) 19-9, a widely known tumour-associated antigen, is useful not only as a diagnostic marker but also as an indicator of prognosis and for monitoring of therapeutic effects in malignant diseases in the pancreato-biliary region. Indeed, elevation of pre-operative CA19-9 (blood CA19-9 levels >37 U/mL) are correlated with the recurrence of intrahepatic cholangiocarcinoma^6^. Moreover, in patients with resectable pancreatic adenocarcinoma, decreased CA19-9 after surgical resection (blood CA19-9 levels ≤200 U/mL) is an independent predictor of patient survival^7^. However, CA19-9 levels show low sensitivity and low specificity because increased in CA19-9 are even observed with nonmalignant diseases that cause obstructive jaundice and inflammatory conditions^8–11^. Carcinoembryonic antigen (CEA) is a similar prognostic factor; however, its accuracy is also inadequate^8,9,12^. Therefore, to improve treatment outcomes, novel biomarkers with high accuracy for the prediction of postoperative recurrence and prognosis in patients with BTC are urgently required.

MicroRNAs (miRNAs) are a class of endogenous noncoding, single-stranded small regulatory RNA molecules involved in post-transcriptional regulation of genes by targeting mRNA^13–15^. MiRNAs have various roles in intracellular biological process, e.g., metabolism, survival, differentiation, and apoptosis^16,17^. Furthermore, many miRNAs have been reported as potential biomarkers of various types of cancers^18–31^. In association with BTC, circulating miRNAs in blood, including *miR-21*^32^, *miR-26a*^33^, *miR-106a*^34^, *miR-192*^35^, and combinations of several miRNAs^36^, have been reported as diagnostic or prognostic biomarkers. Moreover, these miRNAs have been shown to contribute to the proliferation, migration, invasion, and chemoresistance of tumours. Additionally, using miRNA expression profiles of BTC tissue samples extracted from The Cancer Genome Atlas (TCGA) database, Wang *et al*. also showed that six miRNAs (*miR-483-5p*, *miR-675*, *miR-139-3p*, *miR-598*, *miR-625*, and *miR-187*) could serve as predictive markers for the prognosis of patients with BTC^37^. These reports have typically focused on whether specific miRNAs could be useful diagnostic and prognostic markers of all stages of BTC.

In the present study, we investigated the usefulness of circulating miRNA signatures as predictive markers for postoperative recurrence and prognosis in patients with BTC after radical surgery. Our results provided important insights into the use of miRNAs for patient stratification, management, and improvement of prognostic outcomes of treatment in patients with BTC at a resectable stage.

## Results

### Clinical characteristics of patients with BTC after radical surgery with and without recurrence

In total, 66 serum samples were obtained from 22 patients with BTC who underwent curative surgery, including 17 men and five women (average age: 65.4 years [range: 31–81 years]). The median observation period was 1454 days. Of the 22 patients, 13 had recurrence (recurrence group), and nine had no recurrence (nonrecurrence group) during the observation period. The clinical characteristics of patients in the recurrence and nonrecurrence groups are compared in Table 1. There were no significant differences in sex, age, and disease type between the two groups. In pathological TNM staging at diagnosis, there were four patients with stage I cancer and nine patients with stage II cancer in the recurrence group, whereas there were five patients with stage I cancer, three patients with stage II cancer, and one patient with stage III cancer in the nonrecurrence group. The ratio of patients with stage II or higher cancer was relatively higher in the recurrence group than in the nonrecurrence group, although this difference was not significant (*P* = 0.384). Additionally, there were no significant differences in pre-operative CA19-9 or CEA values or in pathological differentiation degree and lymph metastasis between the two groups.

**Table 1:** Clinical characteristics of patients with BTC in the recurrence and nonrecurrence groups.

	Recurrence group	Nonrecurrence group	*P* value
No. of patients	13	9	
Sex (men/women)	10/3	7/2	1.000
Age, years, mean ± SD	67.0 ± 12.8	63.1 ± 9.4	0.448
Disease type			0.648
Bile duct cancer	8 (61.5%)	7 (77.8%)	
Gallbladder cancer	3 (23.0%)	2 (22.2%)	
Ampulla of vater cancer	2 (15.4%)	0 (0%)	
Pathological TNM staging			
(I/II/III/IV)	4/9/0/0	5/3/1/0	0.384
CA19-9 (U/mL)	30.2 ± 32.4	28.6 ± 27.5	0.324
≤37	8 (61.5%)	6 (66.6%)	
>37	5 (38.5%)	3 (33.3%)	
CEA (ng/mL)	30.2 ± 32.4	28.6 ± 27.5	0.117
≤5	8 (61.5%)	6 (66.6%)	
>5	5 (38.5%)	3 (33.3%)	
Pathological differentiation			0.958
Well-differentiated	4 (30.8%)	3 (33.3%)	
Moderately differentiated	7 (53.8%)	5 (55.6%)	
Poorly differentiated	2 (15.4%)	1 (11.1%)	
Lymph metastasis (yes/no)	7/6	3/6	0.415

Abbreviations: BTC, bile tract cancer; SD, standard deviation; CA19-9, carbohydrate antigen 19-9; CEA, carcinoembryonic antigen.

### Extraction of candidate serum miRNAs associated with the prediction of recurrence and prognosis in patients with BTC after radical surgery

**Figure 1:**
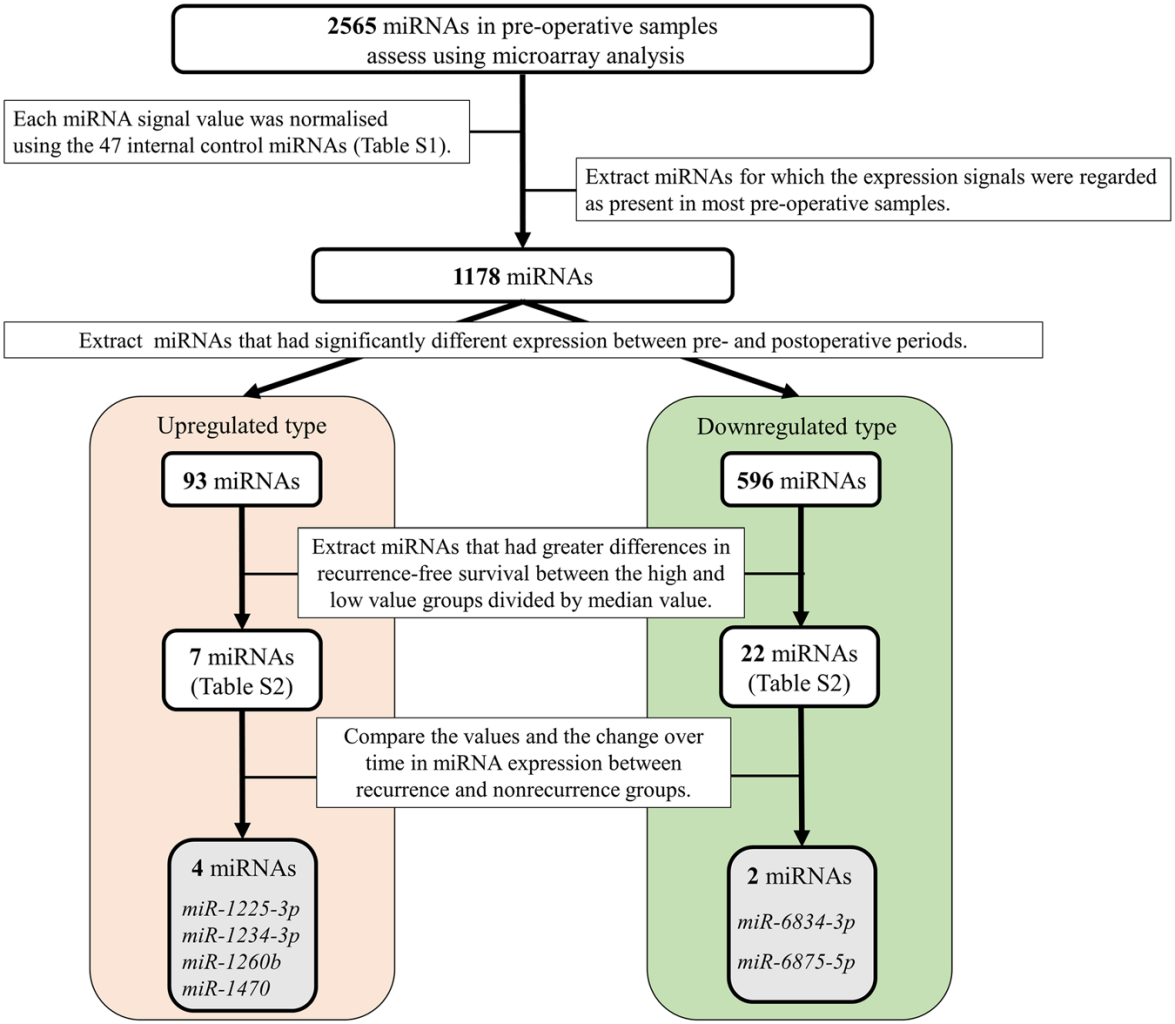
The algorithm to identify multiple candidate miRNAs related to prediction of recurrence and prognosis using pre-operative samples.

Next, we analysed the serum miRNA expression profiles in the blood of patients with BTC and compared the results between the recurrence and nonrecurrence groups. From this analysis, we identified several candidate serum miRNAs associated with the prediction of recurrence and prognosis in patients with BTC after radical surgery. The algorithm is shown in Fig. 1. In total, 2565 miRNAs were analysed by microarray analysis, and the results were normalised using 47 miRNAs with stable expression levels (internal control miRNAs; Supplementary Table S1). Among these miRNAs, we extracted 1178 miRNAs whose expression was detected (expression value >0) for most pre-operative samples. In addition, among the 1178 miRNAs, we selected 689 miRNAs that had significantly different expression between pre-operative and postoperative sample by paired *t* tests. For 93 miRNAs, expression at the pre-operative time point was significantly higher than that at the postoperative time point. In contrast, 596 miRNAs were significantly lower at the pre-operative time point compared with that at the postoperative time point. Among these 93 and 596 miRNAs, we extracted seven and 22 miRNAs (Supplementary Table S2), respectively, that showed significant differences in RFS between the high and low groups divided by the median values using Kaplan-Meier analysis. Finally, evaluating changes in miRNA expression over time at the three time points (pre-operative, postoperative, and recurrence/last observation), we identified six candidate miRNAs as factors related to the prediction of recurrence and prognosis in patients with BTC after radical surgery. Four miRNAs (*miR-1225-3p*, *miR-1234-3p*, *miR-1260b*, and *miR-1470*) were upregulated, and two miRNAs (*miR-6834-3p* and *miR-6875-5p*) were downregulated.

### Comparison of six candidate miRNAs at the pre-operative time point between the recurrence and nonrecurrence groups in patients with BTC after radical surgery

**Figure 2:**
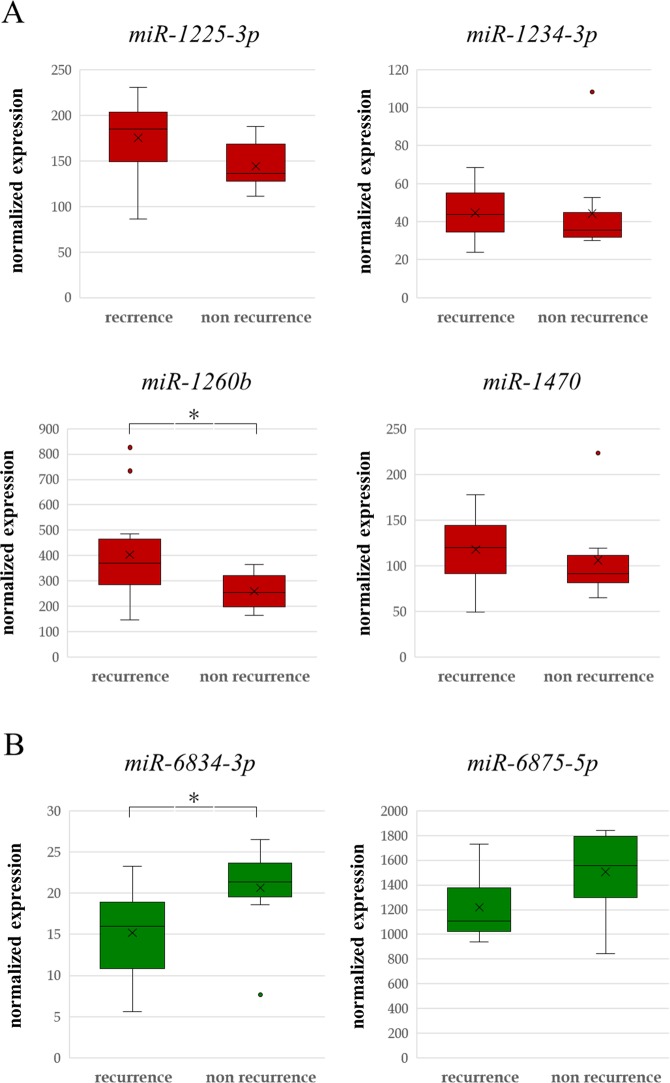
Comparison of the expression of six candidate miRNAs at the pre-operative time point between the recurrence and nonrecurrence groups in patients with BTC after radical surgery. Four miRNAs were upregulated (**A**), and two miRNAs were downregulated (**B**). Two-sided Student’s *t* tests were used to analyse the differences. *P < 0.05.

We then compared the expression of six candidate miRNAs at the pre-operative time point between the recurrence and nonrecurrence groups in patients with BTC after radical surgery. Differences in the expression levels of the six candidate miRNAs at the pre-operative time point between the two groups are shown in Fig. 2. The four upregulated miRNAs were overexpressed in the recurrence group compared with those in the nonrecurrence group (Fig. 2A). The higher expression levels of these four miRNAs at the pre-operative time point may have increased the risk of recurrence. In contrast, the two downregulated miRNAs showed reduced expression in the recurrence group compared with that in the nonrecurrence group (Fig. 2B). Thus, the lower expression levels of these two miRNAs at the pre-operative time point may be related to recurrence.

In addition, we evaluated the expression of six candidate miRNAs at the pre-operative time point between the recurrence and nonrecurrence groups in patients with bile duct cancer, gallbladder cancer, and ampulla vater cancer, respectively (Supplementary Fig. S1). Similar to whole patients with BTC, in patients with bile duct cancer, the four upregulated miRNAs expression levels were higher in the recurrence group (n = 8) than in the nonrecurrence group (n = 7), whereas the two downregulated miRNAs expression levels were lower in the recurrence group (n = 8) than in the nonrecurrence group (n = 7). Also, in comparison between recurrence groups with bile duct cancer (n = 8) and gallbladder cancer (n = 3), of the four upregulated miRNAs, *miR-1260b* were higher expressed in the recurrence group with bile duct cancer than with gallbladder. The other three upregulated miRNAs expression levels were almost equal. In addition, the two downregulated miRNAs were lower expressed in the recurrence group with bile duct cancer than with gallbladder cancer.

### Comparison of changes in the expression levels of six candidate miRNAs at three time points between the recurrence and nonrecurrence groups in patients with BTC after radical surgery

**Figure 3:**
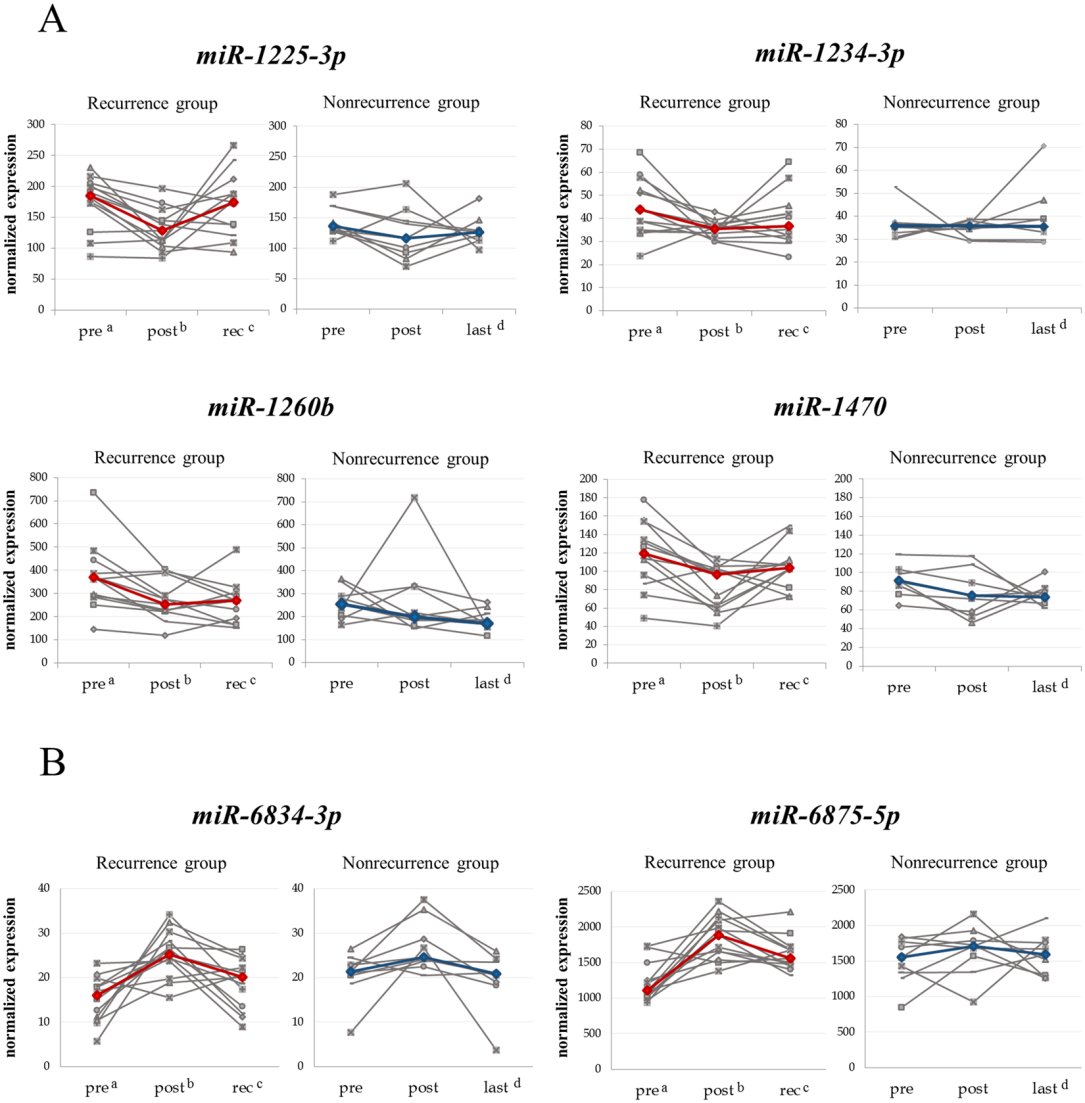
Changes in the expression of six candidate miRNAs at three time points in the recurrence and nonrecurrence groups of patients with BTC after radical surgery. Four miRNAs were upregulated (**A**), and two miRNAs were downregulated (**B**). The grey line indicates the transition in the miRNA expression value of each patient. Red and blue lines show the transitions of median expression values of miRNAs in patients with BTC in the recurrence and nonrecurrence groups, respectively. ^a^pre, pre-operative time point. ^b^post, postoperative time point. ^c^rec, recurrence time point. ^d^last, last observation time point.

Next, we compared changes in the expression levels of the six candidate miRNAs at the three time points between the recurrence and nonrecurrence groups (Fig. 3). In the recurrence group, the expression levels of the upregulated miRNAs (*miR-1225-3p*, *miR-1234-3p*, *miR-1260b*, and *miR-1470*) were decreased at the post-operative time point compared with that in the pre-operative time point in most patients, and elevated expression was then observed at the recurrence time point. In contrast, in the nonrecurrence group, after an initial decrease postoperatively, the expression levels of these miRNAs at the last observation time point were stable or decreased compared with those at the post-operative time point in about half of the patients. In the remaining half of the patients, the expression levels of these miRNAs at the last observation time point increased again; however, the rate of re-elevated miRNA expression was lower than that in the recurrence group (Fig. 3A). Moreover, in the recurrence group, the expression levels of the downregulated miRNAs (*miR-6834-3p* and *miR-6875-5p*) were increased in the post-operative time point compared with those in the pre-operative time point in most patients; the expression levels of these miRNAs then decreased again at the recurrence time point. In contrast, in the nonrecurrence group, after increasing postoperatively, the expression levels of these miRNAs decreased again at the last observation time point in most patients; however, the rate of secondary reduction of miRNA expression was lower than that in patients in the recurrence group (Fig. 3B).

In addition, we evaluated changes in the expression levels of the six candidate miRNAs at the three time points between the recurrence and nonrecurrence groups in patients with bile duct cancer, gallbladder cancer, and ampulla vater cancer, respectively (Supplementary Figs S2–4). In patients with bile duct cancer (n = 15), the changes of all 6 candidate miRNA expression were almost similar to that of whole patients with BTC.

### A discriminant formula for pre-operative expression levels of the six candidate miRNAs and the combination of these miRNAs as predictors of recurrence

Next, we evaluated the sensitivity, specificity, and accuracy of pre-operative levels of the six candidate miRNAs as predictive markers of recurrence using Fisher’s linear discriminant analysis. We calculated the cut-off value for each candidate miRNA and examined whether the expression values of these miRNAs could be predictor for the recurrence of BTC. The discrimination performances of the six candidate miRNAs are presented in Table 2. Notably, for all six candidate miRNAs, we obtained an accuracy of over 70%. In particular, both *miR-1225-3p* and *miR-6875-5p* showed the highest accuracy of 81.8%.

**Table 2:** Discriminant analysis of the six candidate miRNAs as predictive markers of recurrence in patients with BTC.

Discriminant	Accuracy (%)	Sensitivity (%)	Specificity (%)	AUC
***miR-1225-3p***	**81**.**8**	**76**.**9**	**88**.**9**	**0**.**744**
*miR-1234-3p*	72.7	69.2	77.8	0.641
*miR-1260b*	72.7	53.8	100.0	0.803
*miR-1470*	72.7	69.2	77.8	0.675
*miR-6834-3p*	77.2	76.9	77.8	0.821
***miR-6875-5p***	**81**.**8**	**76**.**9**	**88**.**9**	**0**.**778**

Abbreviations: BTC, bile tract cancer; AUC, area under the curve.

We then aimed to achieve higher discriminant performance by combining the six candidate miRNAs. All combinations of the six candidate miRNAs (one or more) were evaluated using Fisher’s linear discriminant analysis. Up to a combination of three candidate miRNAs, the accuracy became higher as the number of miRNAs increased. However, with combinations of four or more miRNAs, the accuracy did not increase further. The combinations of candidate miRNAs showing the best accuracy are listed in Table 3. Two combinations of three miRNAs (*miR-1225-3p*/*miR-1260b*/*miR-6875-5p* and *miR-1260b*/*miR-6834-3p*/*miR-6875-5p*) had the highest accuracy (90.9%), sensitivity (84.6%), and specificity (100.0%). Additionally, the accuracies of pre-operative CEA and CA19-9 values were both 68.2%, which was lower than those of the six candidate miRNAs and their combinations (Table 3).

**Table 3:** Discriminant analysis of combinations of candidate miRNAs showing the highest accuracy for each number of miRNAs used (1–3) as predictive markers of recurrence in patients with BTC.

Discriminant	Accuracy (%)	Sensitivity (%)	Specificity (%)	AUC
*miR-1225-3p*	81.8	76.9	88.9	0.744
*miR-6875-5p*	81.8	76.9	88.9	0.778
*miR-1470*, *miR-6834-3p*	86.4	92.3	77.8	0.829
***miR-1225-3p***, ***miR-1260b***, ***miR-6875-5p***	**90**.**9**	**84**.**6**	**100**.**0**	**0**.**897**
***miR-1260b***, ***miR-6834-3p***, ***miR-6875-5p***	**90**.**9**	**84**.**6**	**100**.**0**	**0**.**880**
CEA value at diagnosis	68.2	76.9	55.6	0.668
CA19-9 value at diagnosis	68.2	76.9	55.6	0.632

Abbreviations: BTC, bile tract cancer; AUC, area under the curve; CEA, carcinoembryonic antigen; CA19-9, carbohydrate antigen 19-9.

### Evaluation of a recurrence predictive index created from the combination of candidate miRNAs

**Figure 4:**
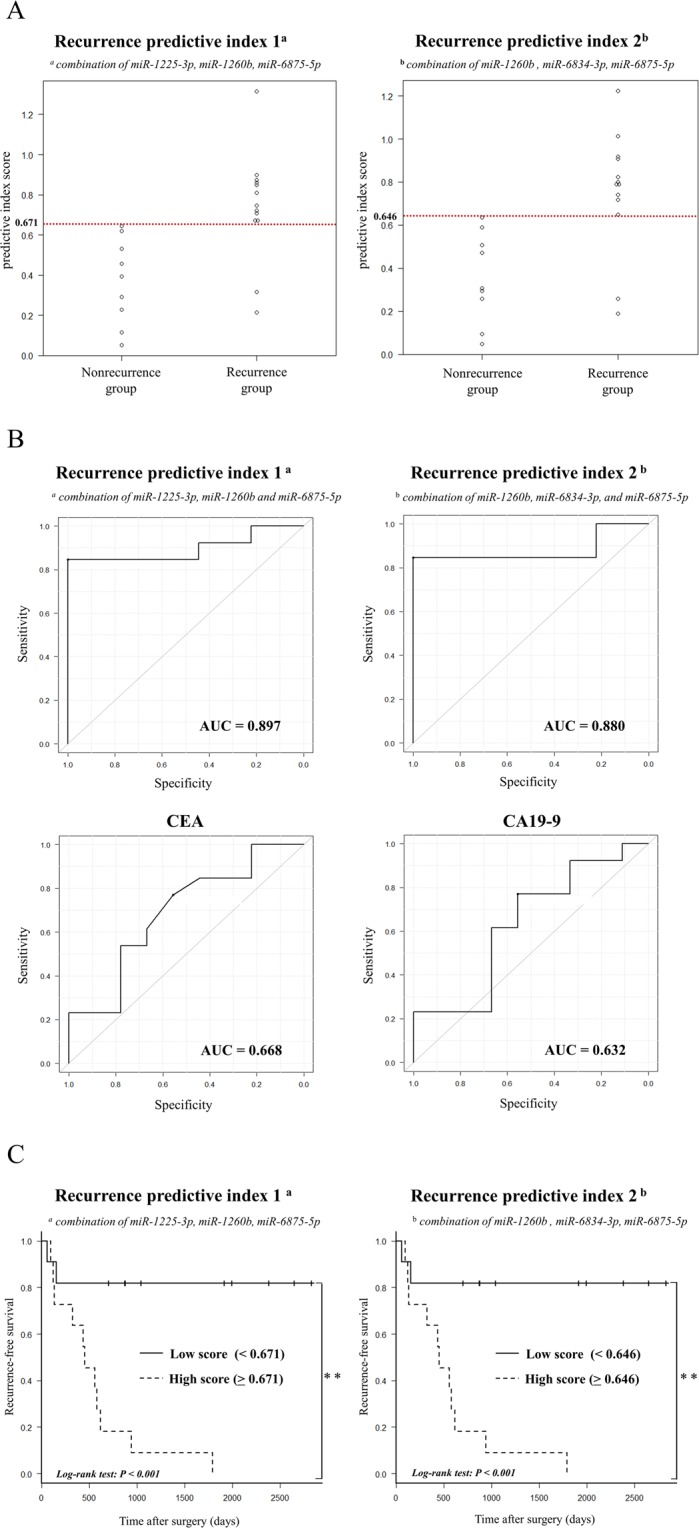
Evaluation of our novel indices using a combination of candidate miRNAs as a recurrence prediction marker in patients with BTC after radical surgery. (**A**) Comparison of recurrence predictive index scores between the recurrence and nonrecurrence groups. (**B**) Receiver operator characteristic (ROC) curve analysis of recurrence predictive indices in comparison with CEA and CA19-9 values. Area under the curve (AUC) values are shown on the graphs. (**C**) Association of recurrence predictive indices with recurrence-free survival (RFS). Kaplan-Meier graphs representing the probabilities of RFS in the enrolled patients according to recurrence predictive index scores. Log-rank tests were used to analyse the significance of the differences. **P < 0.001.

We then created two recurrence predictive indices from combinations of three candidate miRNAs with the highest accuracies. We defined the recurrence predictive index created from *miR-1225-3p*, *miR-1260b*, and *miR-6875-5p* as index 1, which was calculated using the following formula: (0.238 × *miR-1225-3p*) + (0.320 × *miR-1260b*) – (0.473 × *miR-6875-5p*) + 1.09. Additionally, we defined the recurrence predictive index created from *miR-1260b*, *miR-6834-3p*, and *miR-6875-5p* as index 2, which was calculated using the following formula: (0.321 × *miR-1260b*) – (0.216 × *miR-6834-3p*) – (0.421 × *miR-6875-5p*) + 3.14. A comparison these index scores between the recurrence and nonrecurrence groups is presented in Fig. 4A. The cut-off value in the index 1 was 0.671. In the nonrecurrence group, the index 1 score was lower than the cut-off value in all patients. In contrast, in the recurrence group, the index 1 score was higher than the cut-off value in most patients. Therefore, we demonstrated that patients with BTC with higher index 1 scores (≥cut-off value) at the pre-operative period had a greater risk of developing recurrence, whereas in patients with BTC with lower index 1 scores (<cut-off value) at the pre-operative time point, the risk of recurrence was lower. We obtained similar results for index 2, for which of the cut-off value was 0.646. In addition, for two patients in the recurrence group with lower index 1 and 2 scores than the cut-off value, recurrence developed within a relatively short period after radical surgery (57 and 154 days, respectively). Thus, these findings suggested that these indices could be useful as predictive markers of long-term recurrence (e.g., more than half a year).

We then compared the usefulness of CEA and CA19-9 as predictive markers of recurrence predictive using receiver operating characteristic (ROC) curves (Fig. 4B). We found that indices 1 and 2 were more effective as predictive markers because the area under the curve (AUC) values of these indices were much higher than those of CEA and CA19-9. In addition, even for the ROC curves of the six candidate miRNAs, the AUCs were higher for most candidate miRNAs compared with those of CEA and CA 19-9 (Supplementary Fig. S5).

### Association of recurrence predictive index scores with RFS in patients with BTC after radical surgery

In the two recurrence predictive indices of candidate miRNAs, we divided the enrolled patients into two groups using the cut-off values and analysed RFS using Kaplan-Meier analysis (Fig. 4C). For both indices, recurrence was significantly more likely in the high index score group (≥cut-off value) than in the low index score group (<cut-off value; *P* < 0.001). In addition, we analysed the RFS for the six candidate miRNAs using Kaplan-Meier analysis (Supplementary Fig. S6). The results showed that for the four upregulated miRNAs (*miR-1225-3p*, *miR-1234-3p*, *miR-1260b*, and *miR-1470*), relapse was more likely in the high score group than in the low score group. Conversely, for the two downregulated miRNAs (*miR-6834-3p* and *miR-6875-5p*), relapse was more likely in the low score group than in the high score group. For the association of pre-operative CEA (cut-off, 1.9 U/mL) and CA19-9 values (cut-off, 30.4 U/mL) with RFS, there were no statistically significant differences between the high and low value groups, although there was a tendency to relapse more easily in the high value group (Supplementary Fig. S9).

### Association of the recurrence predictive index score with OS in patients with BTC after radical surgery

Similar to RFS, we analysed OS using Kaplan-Meier analysis. For both indices, patients in the low index score group (≥ cut-off value) had significantly better prognosis than those in the high index score group (< cut-off value; *P* < 0.001; Supplementary Fig. S7). For the four upregulated miRNAs (*miR-1225-3p*, *miR-1234-3p*, *miR-1260b*, and *miR-1470*), patients in the low score group tended to have better prognosis than those in the high score group. Conversely, for the two downregulated miRNAs (*miR-6834-3p* and *miR-6875-5p*), patients in the high score group tended to have better prognosis than those in the low score group (Supplementary Fig. S8). However, for all six of the candidate miRNAs, no significant differences were observed between the high and low score groups. Analysis of the association of pre-operative CEA (cut-off, 1.9 U/mL) and CA19-9 values (cut-off, 30.4 U/mL) with OS showed that there were no statistically significant differences between the high and low value groups, although there was a tendency for a worse prognosis in the high value group (Supplementary Fig. S9).

### Clinical factors related to RFS in patients with BTC after radical surgery

Univariate and multivariate analyses using the Cox proportional hazards model were performed to evaluate clinical factors related to RFS (Table 4). In univariate analysis, recurrence predictive indices 1 and 2 were significantly associated with RFS. Furthermore, in multivariate analyses of factors with hazard ratios (HRs) of 2 or more in univariate analysis, we demonstrated that index 1, index 2, and lymph metastasis were independent significant factors related to RFS (*P* = 0.002, *P* = 0.002, *P* = 0.006, respectively).

**Table 4:** Univariate and multivariate analyses of clinical factors related to recurrence-free survival in patients with BTC after surgery.

	Univariate			Multivariate		
	HR	95% CI	*P* value	HR	95% CI	*P* value
Recurrence predictive index 1^a^	**9**.**711**	**2**.**085–45**.**24**	**0**.003*	**15**.**76**	**2**.**584–95**.**15**	**0**.002*
Recurrence predictive index 2^b^	**9**.**711**	**2**.**085–45**.**24**	**0**.003*	**15**.**76**	**2**.**584–95**.**15**	**0**.002*
Age at diagnosis	1.022	0.970–1.076	0.412			
Sex (men/women)	1.179	0.321–4.336	0.804			
Disease type of BTC	1.468	0.704–3.059	0.306			
Pathological stage at diagnosis	1.512	0.631–3.624	0.354			
Pathological differentiation	1.542	0.614–3.875	0.357			
Lymph metastasis (yes/no)	2.648	0.869–8.069	0.087	**8**.**884**	**1**.**844–42**.**80**	**0**.006*
Pre-operative CA19–9 value	2.444	0.734–8.143	0.146	2.656	0.596–11.83	0.200
Pre-operative CEA value	2.682	0.736–9.778	0.135	2.358	0.552–10.06	0.246

^a^Index extracted by the combination of *miR-1225–3p*, *miR-1260b*, and *miR-6875–5p*.

^b^Index extracted by the combination of *miR-1260b*, *miR-6834–5p*, and *miR-6875–5p*.

*Significant relationship between clinical parameters and overall survival.

Abbreviations: BTC, bile tract cancer; HR, hazard ratio; 95% CI, 95% confidence interval; CA19–9, carbohydrate antigen 19–9; CEA, carcinoembryonic antigen.

### Clinical factors related to OS in patients with BTC after radical surgery

Similar to RFS, we then evaluated the clinical factors related to OS (Supplementary Table S3). In univariate analysis, we showed that index 1, index 2, and lymph metastasis were significantly associated with OS. Moreover, multivariate analyses showed that index 1 and index 2 were independent significant factors related to OS (*P* = 0.022, *P* = 0.022, respectively).

## Discussion

Detection of early-stage BTC is extremely difficult^38–42^, and even when radical surgery is carried out at early stages of the disease, recurrence or metastasis is still frequently observed^4,5,43^. Furthermore, in cases with recurrence or metastasis of BTC, there are no effective standard therapies after the second treatment, and the prognosis is quite poor^3,39,40^. Accordingly, prediction of recurrence of BTC using pre-operative examinations may help prevent recurrence and metastasis through the development of novel management strategies, including pre- and postoperative adjuvant chemotherapies. Therefore, the identification of novel, effective biomarkers to adequately predict recurrence and prognosis is urgently required to improve life prognosis, while maintaining good quality of life in patients with BTC after radical surgery.

CA19-9 and CEA have often been used as diagnostic and prognostic markers for patients with BTC. However, the accuracies of these biomarkers as prognostic markers are often low because the values of these markers depend on various clinical factors, including the presence of other cancers and nonmalignant diseases, such as obstructive jaundice and inflammation^8–12^. In this study, our results demonstrated that the usefulness of CA19-9 and CEA as predictive markers of recurrence and prognosis in patients with BTC after radical surgery was unclear. A simple alternative predictive marker of recurrence and prognosis, which can be assessed using a noninvasive blood test, is necessary to improve outcomes in patients with BTC who have undergone radical surgery.

In recent years, many studies have examined the usefulness of serum miRNAs in several types of cancers^18–36^. Serum miRNAs can be evaluated more quickly and less invasively than miRNAs from tissue samples, thereby reducing the burden on patients. Moreover, serum miRNAs, which are secreted from cells and tissues, can specifically reflect the state of the tumour itself or the tumour microenvironment^44–48^. Several evidences showed that many extracellular miRNAs have been contained within the exosome which is small membrane vesicles of endocytosis origin secreted by most cell types, and they could play an important purpose in intercellular communications; thus, exosomal miRNAs, which have been wrapped in vesicles, may not be degraded by RNA-decomposing enzymes and could be relatively stable, permitting long-term storage and re-analysis of samples^49–53^. Indeed, it has been demonstrated that the majority of miRNAs in body fluids are present into exosomes^54^. Also, Umezu *et al*. reported that some exosomal miRNAs could play an important role in leukemia cell-to-endothelial cell communication, which may be, in part, associated with angiogenic activity in endothelial cells^55^. In addition, Sueta *et al*. showed that several exosomal miRNAs might be useful biomarkers to predict breast cancer recurrence^56^. Whereas, Recent reports suggested that most of circulating miRNAs independently exist not only into exosomes, but also binding to the Ago2 protein, which is a part of the RNA-induced silencing complex^57,58^. In addition, Arroyo *et al*. found that the Ago2-binding miRNAs were derived from cell types different from exosomal miRNAs^57^. Other circulating miRNAs, including the Ago2-binding miRNAs, also could influence cell-cell communication. In this study, we evaluated the expression of whole serum miRNA, including exosomal and Ago2-binding miRNAs etc. The examination of the expression of whole serum miRNA is noninvasive and simple, and therefore they could be more excellent for being widely used as diagnostic and prognostic markers and as potential therapeutic targets, compared to other methods such as measuring exosome.

A number of new cancer-related miRNAs have recently been identified; however, most of these miRNAs have not been assessed with regard to their relationship to recurrence and prognosis in patients with BTC, particularly in those who have undergone radical surgery. In this study, we evaluated the expression profiles of many serum miRNAs in patients with BTC after radical surgery using highly sensitive microarrays, permitting the simultaneous analysis of more than 2500 miRNAs that were recently updated in miRBase (release 21). We compared expression values of serum miRNAs in the recurrence and nonrecurrence groups of patients with BTC after radical surgery. Our study followed the post-operative outcomes of patients who underwent radical surgery over a long time period; the median observation period was 1454 days. In addition, in the all enrolled patients, we could examine expression values of serum miRNAs at three time points (before surgery, after surgery, and recurrence/last observation) and clarified the changes in miRNA levels at the three time points; to the best of our knowledge, such a comprehensive study has not been performed previously.

From our analysis, we identified six miRNAs related to the recurrence and prognosis of BTC in patients who had undergone radical surgery, according to our newly developed algorithm based on changes in miRNA expression. Among the six identified miRNAs, *miR-1225-3p* and *miR-6875-5p*, which were single miRNAs identified as recurrence predictive markers, had the best accuracy of 81.8%. Furthermore, by combining multiple significant miRNAs, we obtained a more robust recurrence predictive ability, as previously demonstrated^59,60^. As a result, by combining three of the six candidate miRNAs, we finally achieved the best discriminant performance in predicting the recurrence of BTC after radical surgery (sensitivity: 84.6%, specificity: 100.0%, accuracy: 90.9%). When more miRNAs were combined, the discrimination performance did not improve, potentially related to the occurrence of noise. This phenomenon is known as the “curse of dimensionally” in algorithm development^61^.

Next, we created novel recurrence predictive indices from combination of the three miRNAs described above. Our results demonstrated the clinical value of these indices; the indices could be useful to predict the recurrence of BTC after radical surgery at the pre-operative time point in most enrolled patients. Both of the patients in whom recurrence could not be predicted had gallbladder cancer and lymph node metastasis and relapsed within a relatively short time. Thus, our novel recurrence predictive indices may be effective for predicting the recurrence of BTC after radical surgery over a long-term period, particularly from 6 months to 5 years after surgery. In addition, we demonstrated that patients with low index scores had significantly better RFS compared with those with higher index scores, and these indices were independent factors related to RFS. Therefore, our findings suggested that these novel recurrence predictive indices could be useful as predictive markers of recurrence and prognosis in patients with BTC after radical surgery. In the future, measurement of these candidate serum miRNAs before surgery and evaluation of the novel indices described herein may help to stratify patients with BTC according to the risk of recurrence and could lead to improvements in prognosis via new approaches to disease management and adjuvant chemotherapy. Moreover, our results also showed that lymph node metastasis was an independent factor related to RFS. Thus, further evaluations are necessary to determine whether the accuracy of predicting the recurrence of BTC after radical surgery could be further improved by simultaneously evaluating our novel indices and clinical information, including lymph node metastasis.

Although we found that a combination of serum miRNAs could be used as a predictive marker of recurrence and prognosis in patients with BTC after radical surgery, the biological roles of the identified serum miRNAs are still unclear. All six candidate miRNAs that we identified had ID names higher than 1,000, indicating that they were discovered relatively recently. Therefore, there are only a few reports describing the mechanisms through which these miRNAs are involved in various cancers or chronic diseases that cause cancers (see Supplementary Table S4)^62–69^. We could predict a total 884 target genes of six candidate miRNAs by TargetScan Human 7.2 software and Mirtarbase database. The interaction network of these miRNAs and target genes are shown in Supplementary Fig. S10. We found that all 6 candidate miRNAs had conglomerate target genes, and several target genes overlapped among these miRNAs. In addition, the enrichment and function analysis of the target genes of these miRNAs, including Gene Ontology (GO) biological processes terms and Kyoto Encyclopedia of Genes and Genomes (KEGG) pathways, were performed basing on database for annotation visualization and integrated discovery (DAVID) database (Supplementary Fig. S11). In upregulated miRNAs, the most significant enriched GO term and KEGG pathway were protein binding and signaling pathways regulating pluripotency of stem cells, respectively. Also, in downregulated miRNAs, the most significant enriched GO term and KEGG pathway were protein binding and endocytosis, respectively. Recently, Wardell *et al*. reported identification of some driver genes and the predisposing gene mutations of BTC by large-scale genome sequence analysis in patients with BTC^70^. We believe that additional studies using specimens collected in this study are necessary to more clearly elucidate the biological pathway of the identified miRNAs.

For the six candidate miRNAs identified in this study, we evaluated the expression levels of these miRNAs in tissue samples of bile duct cancer using miRNA expression profiles extracted from TCGA database. The results showed that in tissue samples from bile duct cancer, there were no major differences in the expression values of all six candidate miRNAs compared with those in healthy tissue^37^. Therefore, the differences between our findings in this study and the results from tissue expression in TCGA database suggest that the underlying mechanisms of circulating miRNAs could be more complex than previously speculated, including the exosome secretion theory. In our study, because most of the resected tumour samples from the enrolled patients were not large lumps but developed horizontally along the bile duct, we were not able to obtain sufficient tissue samples to examine the expression levels of miRNAs. In future studies, it will be necessary to investigate the expression levels of these candidate miRNAs in tissue samples of BTC after radical surgery and to compare the results between recurrence and nonrecurrence groups.

In conclusion, our results clarified that the six candidate miRNAs as well as combinations of these miRNAs could be applied as predictive markers of recurrence and prognosis in patients with BTC after radical surgery. The usefulness of our novel indices as predictive markers of recurrence and prognosis should be further validated in a large-scale, multicentre study. In the future, we hope that our results will lead to the development of treatment strategies and improvement of life prognosis in patients with BTC after radical surgery.

## Materials and Methods

### Patients and samples

Twenty-six patients with BTC underwent surgical resection at National Cancer Center Hospital East (NCCE) from June 2008 to April 2012. Patients did not have (1) distant metastasis or unresectable advanced BTC, (2) medicinal treatment for cancer before or after surgery, (3) a histologically special tissue type other than adenocarcinoma, or (4) histologically residual disease after surgery. Subsequently, we excluded the following cases: two patients who did not provide informed consent, one patient who was also found to have lung cancer, and one patient who dropped out during postoperative follow-up and therefore did not provide blood samples for this time point. Finally, 22 patients with BTC who had undergone radical surgery were enrolled in this study. All of the enrolled patients were diagnosed histologically with BTC by pathologists, as follows: 15 patients with bile duct cancer, five patients with gallbladder cancer, and two patients with ampulla vater cancer.

We obtained serum samples from 22 enrolled patients at three time points: before radical surgery, after radical surgery, and at the time point of recurrence. In cases without relapse, the time of the last observation period was considered the final time point. All peripheral blood samples were processed to serum within 1 day and stored at −80 °C.

The present study involving human subjects was approved by the NCCE Institutional Review Board (approval no. 2007-060). Written informed consent was obtained from each participant. All samples were collected after obtaining written comprehensive informed consent from the patients. All experiments were performed in accordance with relevant guideline and regulations.

### Evaluation of miRNA expression by microarray analysis

Total RNA was extracted from 300-μL serum samples using a 3D-Gene RNA Extraction Reagent From a Liquid Sample Kit (Toray Industries, Inc., Tokyo, Japan). Comprehensive miRNA expression analysis was performed using a 3D-Gene miRNA Labeling Kit and a 3D-Gene Human miRNA Oligo Chip (Toray Industries, Inc.), which was designed to detect 2565 miRNAs sequences registered in miRBase release 21 (http://www.mirbase.org/).

Each miRNA was regarded as present if the corresponding microarray signal was more than the mean signal of the negative controls, of which the top and bottom 5% ranked by signal intensity were removed. Once the miRNA was considered present, the mean signal of the negative controls was subtracted from the corresponding miRNA signal. In addition, in order to normalise the signals across the different microarrays tested, quantile normalisation was performed. In this study, we used 47 preselected miRNAs as internal control miRNAs; these miRNAs had been stably detected in all serum samples of enrolled patients with BTC (Supplementary Table S1). Each miRNA signal value was standardied to the ratio of the average signal value of these internal control miRNAs to the pre-set value. All microarray data in this study were consistent with the Minimum Information About a Microarray Experiment (MIAME) guidelines and are publicly available through the GEO database (GSE119892, http://www.ncbi.nlm.nih.gov/projects/geo/).

### Statistical analysis

All analyses were performed using R version 3.5.1 (R Foundation for Statistical Computing, http://www.R-project.org). Two-sided Student’s t tests were used to compare continuous variables, and Fisher’s exact tests were used to compare categorical variables. Results with *P* values of less than 0.05 were considered significant. Fisher’s linear discriminant analysis was performed to create recurrence predictive index. ROC analysis and AUC values were used to evaluate recurrence predictive ability. Recurrence and survival analyses were performed using the Kaplan-Meier method. Comparisons were performed using log-rank tests, and univariate and multivariate analyses were performed using a Cox proportional hazards model.

## Supplementary information

SUPPLEMENTARY INFORMATION

## References

[1] Ishihara S (2016). Biliary tract cancer registry in Japan from 2008 to 2013. J. Hepatobiliary Pancreat. Sci..

[2] Siegel RL, Miller KD, Jemal A (2017). Cancer statistics, 2017. CA Cancer J..

[3] Farley DR, Weaver AL, Nagorney DM (1995). ‘Natural history’ of unresected cholangiocarcinoma: patient outcome after noncurative intervention. Mayo Clin. Proc..

[4] Choi SB (2015). Disease recurrence patterns and analysis of clinicopathological prognostic factors for recurrence after resection for distal bile duct cancer. Am. Surg..

[5] Jarnagin WR (2001). Staging, resectability, and outcome in 225 patients with hilar cholangiocarcinoma. Ann. Surg..

[6] Miwa S (2006). Predictive factors for intrahepatic cholangiocarcinoma recurrence in the liver following surgery. J. Gastroenterol..

[7] Ferrone CR (2006). Perioperative CA19-9 levels can predict stage and survival in patients with resectable pancreatic adenocarcinoma. J. Clin. Oncol..

[8] Kau SY (1999). Diagnostic and prognostic values of CA 19–9 and CEA in periampullary cancers. J. Am. Coll. Surg..

[9] Strom BL (1990). Serum CEA and CA 19–9: potential future diagnostic or screening tests for gallbladder cancer?. Int. J. Cancer..

[10] Ballehaninna UK (2012). The clinical utility of serum CA 19–9 in the diagnosis, prognosis and management of pancreatic adenocarcinoma: an evidence based appraisal. J. Gastrointest. Oncol..

[11] Morris-Stiff G (2012). Ca19-9 and pancreatic cancer: is it really that good?. Gastrointest. Oncol..

[12] Wang Y, Yang H, Shen C, Luo J (2015). Cholangiocarcinoma: prognostic factors after surgical resection in China. Int. J. Clin. Exp. Med..

[13] Calin GA, Croce CM (2006). MicroRNA-cancer connection: the beginning of a new tale. Cancer Res..

[14] Bartel DP (2004). MicroRNAs: genomics, biogenesis, mechanism, and function. Cell.

[15] Ambros V (2003). MicroRNA pathways in flies and worms: growth, death, fat, stress, and timing. Cell.

[16] He J (2016). Analysis of miRNAs and their target genes associated with lipid metabolism in duck liver. Sci. Rep..

[17] Thomou T (2017). Adipose-derived circulating miRNAs regulate gene expression in other tissues. Nature.

[18] Hwang S (2018). Differential expression of miRNA199b-5p as a novel biomarker for sporadic and hereditary parathyroid tumors. Sci. Rep..

[19] Kahraman M (2018). MicroRNA in diagnosis and therapy monitoring of early-stage triple-negative breast cancer. Sci. Rep..

[20] Kawaguchi T (2017). Overexpression of suppressive microRNAs, miR-30a and miR-200c are associated with improved survival of breast cancer patients. Sci. Rep..

[21] Chen X (2008). Characterization of microRNAs in serum: A novel class of biomarkers for diagnosis of cancer and other diseases. Cell Res..

[22] Calin GA, Croce CM (2006). MicroRNA signatures in human cancers. Nat. Rev. Cancer.

[23] He L (2005). A microRNA polycistron as a potential human oncogene. Nature.

[24] Calin GA (2005). Frequent deletions and down-regulation of micro-RNA genesmiR15 and miR16 at 13q14 in chronic lymphocytic leukemia. Proc. Natl. Acad. Sci. USA.

[25] Wang LG (2012). Serum microRNA-29a is a promising novel marker for early detection of colorectal liver metastasis. Cancer Epidemiol..

[26] Ng EK (2009). Differential expression of microRNAs in plasma of patients with colorectal cancer: a potential marker for colorectal cancer screening. Gut.

[27] Cummins JM (2006). Implications of micro-RNA profiling for cancer diagnosis. Oncogene.

[28] Wang C (2015). A panel of five serum miRNAs as a potential diagnostic tool for early-stage renal cell carcinoma. Sci. Rep..

[29] Yanaihara N (2006). Unique microRNA molecular profiles in lung cancer diagnosis and prognosis. Cancer Cell.

[30] Motawi TK (2016). Circulating microRNAs, miR-92a, miR-100 and miR-143, as non-invasive biomarkers for bladder cancer diagnosis. Cell. Biochem. Funct..

[31] Bertoli G (2015). New biomarkers for diagnosis, prognosis, therapy prediction and therapeutic tools for breast cancer. Theranostics.

[32] Kishimoto T (2013). Plasma miR-21 is a novel diagnostic biomarker for biliary tract cancer. Cancer Sci..

[33] Wang LJ (2015). Serum miR-26a as a diagnostic and prognostic biomarker in cholangiocarcinoma. Oncotarget.

[34] Cheng Q (2015). Circulating miR-106a is a novel prognostic and lymph node metastasis indicator for cholangiocarcinoma. Sci. Rep..

[35] Silakit R (2014). Circulating miR-192 in liver fluke-associated cholangiocarcinoma patients: a prospective prognostic indicator. J. Hepatobiliary. Pancreat. Sci..

[36] Kojima M (2015). MicroRNA markers for the diagnosis of pancreatic and biliary-tract cancers. PLoS One.

[37] Wang M (2015). A six-microRNA set as prognostic indicators for bile duct cancer. Int. J. Clin. Exp. Med..

[38] Mathema VB (2015). Current insights on cholangiocarcinoma research: a brief review. *Asian Pacific J*. *Cancer*. Prevent..

[39] Aljiffry M (2009). Advances in diagnosis, treatment and palliation of cholangiocarcinoma: 1990–2009. World J. Gastroenterol..

[40] Khan SA (2007). Cholangiocarcinoma and its management. Gut.

[41] Wu Q (2007). Therapeutic effect of rapamycin on gallbladder cancer in a transgenic mouse model. Cancer Res..

[42] Lin W (2012). Vascular endothelial growth factor-d promotes growth, lymphangiogenesis and lymphatic metastasis in gallbladder cancer. Cancer Lett..

[43] Patel T (2006). Cholangiocarcinoma. Nat. Clin. Pract. Gastroenterol. Hepatol..

[44] Challagundla KB (2013). MicroRNAs in the tumor microenvironment: solving the riddle for a better diagnostics. Expert Rev. Mol. Diagn..

[45] Ling H (2013). MicroRNAs and other non-coding RNAs as targets for anticancer drug development. Nat. Rev. Drug Disc..

[46] Pinweha P (2016). MicroRNAs and oncogenic transcriptional regulatory networks controlling metabolic reprogramming in cancers. Comput. Struct. Biotechnol. J..

[47] Moldovan L (2014). Methodological challenges in utilizing miRNAs as circulating biomarkers. J. Cell. Mol. Med..

[48] Khoury S, Tran N (2009). Circulating microRNAs: potential biomarkers for common malignancies. Biomark. Med..

[49] Kosaka N (2013). Trash or treasure: extracellular microRNAs and cell-to-cell communication. Front. Genet..

[50] Gallo A (2012). The majority of microRNAs detectable in serum and saliva is concentrated in exosomes. PLoS One.

[51] Valadi H (2007). Exosome-mediated transfer of mRNAs and microRNAs is a novel mechanism of genetic exchange between cells. Nat. Cell Biol..

[52] Thery C (2002). Exsosome: composition, biogenesis and function. Nat. Rev. Immunol..

[53] Stoorvogel W (2002). The biogenesis and functions of exsosomes. Traffic.

[54] Gallo A, Tandon M, Alevizos I, Illei GG (2012). The majority of microRNAs detectable in serum and saliva is concentrated in exosomes. PLoS One.

[55] Umezu T, Ohyashiki K, Kuroda M, Ohyashiki JH (2013). Leukemia cell to endothelial cell communication via exosomal miRNAs. Oncogene.

[56] Sueta A (2017). Differential expression of exosomal miRNAs between breast cancer patients with and without recurrence. Oncotarget.

[57] Arroyo JD (2011). Argonaute2 complexes carry a population of circulating microRNAs independent of vesicles in human plasma. Proc Natl Acad Sci USA.

[58] Turchinovich A, Weiz L, Langheinz A, Burwinkel B (2011). Characterization of extracellular circulating microRNA. Nucleic Acids Res.

[59] Taguchi Y, Murakami Y (2013). Principal component analysis based feature extraction approach to identify circulating microRNA biomarkers. PLoS One.

[60] Sato F (2011). MicroRNA profile predicts recurrence after resection in patients with hepatocellular carcinoma within the Milan criteria. PLoS One.

[61] Lee G (2008). Investigating the efficacy of nonlinear dimensionality reduction schemes in classifying gene and protein expression studies. IEEE/ACM Trans. Comput. Biol. Bioinf..

[62] Cheng M (2018). Interferon down-regulation of miR-1225-3p as an antiviral mechanism through modulating Grb2-associated binding protein 3 expression. J. Biol. Chem..

[63] Dong L (2015). Interference with the β-catenin gene in gastric cancer induces changes to the miRNA expression profile. Tumour Biol..

[64] Xu L (2018). MiR-1260b promotes the migration and invasion in non-small cell lung cancer via targeting PTPRK. Pathol. Res. Pract..

[65] Li X, Song H, Liu Z, Bi Y (2018). miR-1260b promotes cell migration and invasion of hepatocellular carcinoma by targeting the regulator of G-protein signaling 22. Biotechnol. Lett..

[66] Hirata H (2014). Genistein downregulates onco-miR-1260b and upregulates sFRP1 and Smad4 via demethylation and histone modification in prostate cancer cells. Br. J. Cancer..

[67] Hirata H (2013). Genistein downregulates onco-miR-1260b and inhibits Wnt-signalling in renal cancer cells. Br. J. Cancer..

[68] Mei LL, Qiu YT, Wang WJ, Bai J, Shi ZZ (2017). Overexpression of microRNA-1470 promotes proliferation and migration, and inhibits senescence of esophageal squamous carcinoma cells. Oncol. Lett..

[69] Nie W (2015). miR-1470 mediates lapatinib induced p27 upregulation by targeting c-jun. J. Cell Physiol..

[70] Wardell CP (2018). Genomic characterization of biliary tract cancers identifies driver genes and predisposing mutations. J Hepatol.

