# Supplementary material for: Usefulness of serum microRNA as a predictive marker of recurrence and prognosis in biliary tract cancer after radical surgery
Source: Sci Rep. 2019 Apr 11;9:5925. doi: 10.1038/s41598-019-42392-7 (PMC6459925; doi:10.1038/s41598-019-42392-7)
Supplement: Supplementary file 1 — SUPPLEMENTARY INFORMATION [file 41598_2019_42392_MOESM1_ESM.pdf]

SUPPLEMENTARY INFORMATION

Usefulness of serum microRNA as a predictive marker of recurrence and prognosis in biliary tract cancer after radical surgery

Yu Akazawa[1,2], Shoichi Mizuno[1], Norihiro Fujinami[1], Toshihiro Suzuki[1], Yusuke Yoshioka[3], Takahiro Ochiya[3,4], Yasunari Nakamoto[2], Tetsuya Nakatsura[1]

[1)]Division of Cancer Immunotherapy, Exploratory Oncology Research and Clinical Trial Center, National Cancer Center
[2)]Second Department of Internal Medicine, Faculty of Medical Sciences, University of Fukui
[3)]Division of Molecular and Cellular Medicine, National Cancer Center Research Institute
[4)]Institute of Medical Science, Tokyo Medical University

Corresponding Author: Tetsuya Nakatsura
Division of Cancer Immunotherapy, Exploratory Oncology Research and Clinical Trial Center,
National Cancer Center, 6-5-1 Kashiwanoha, Kashiwa 277-8577, Japan
E-mail: tnakatsu@east.ncc.go.jp

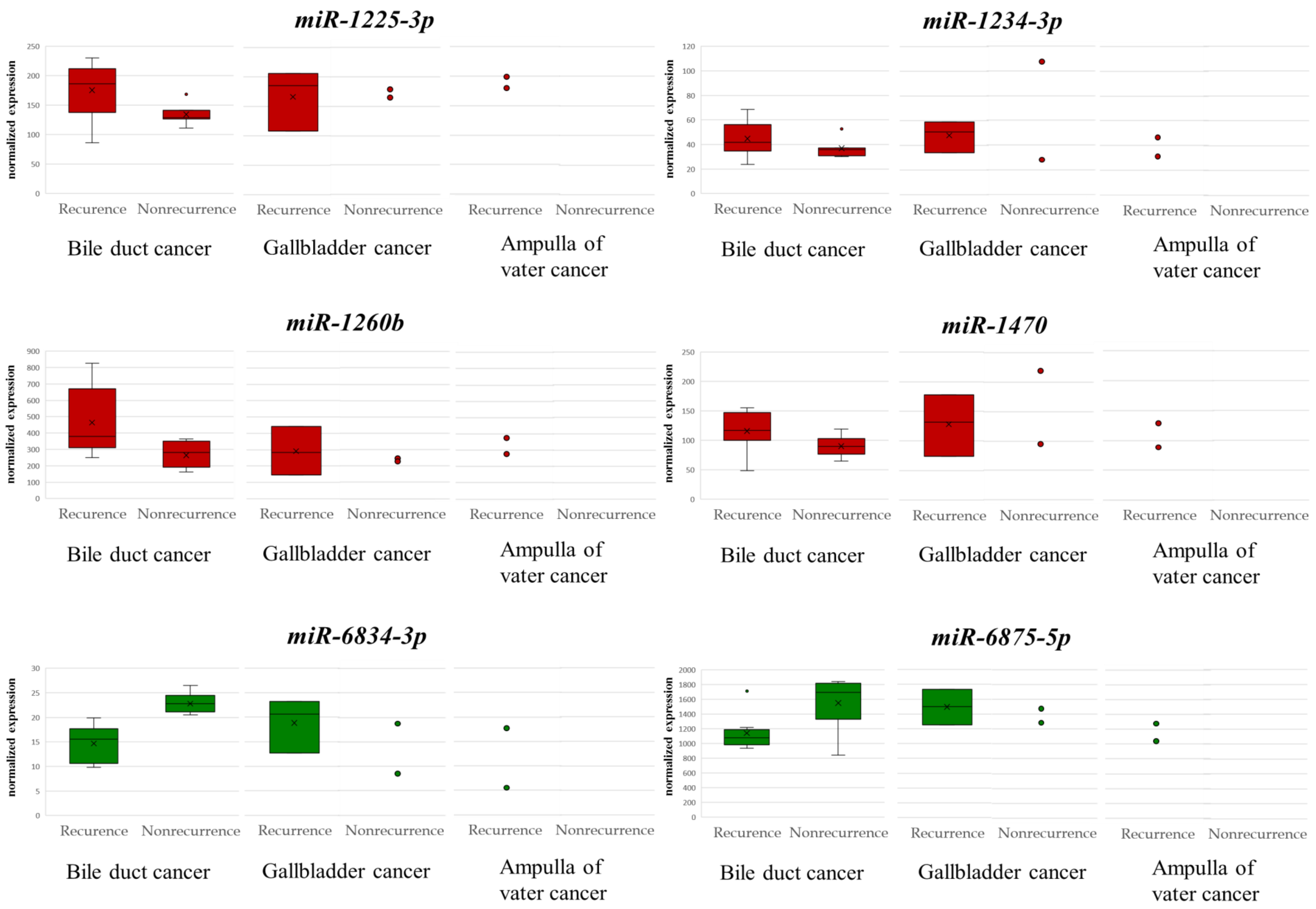

Supplementary Figure S1: The expression of six candidate miRNAs at the pre-operative time point between the recurrence and nonrecurrence groups in patients with bile duct cancer, gallbladder cancer, and ampulla vater cancer, respectively.

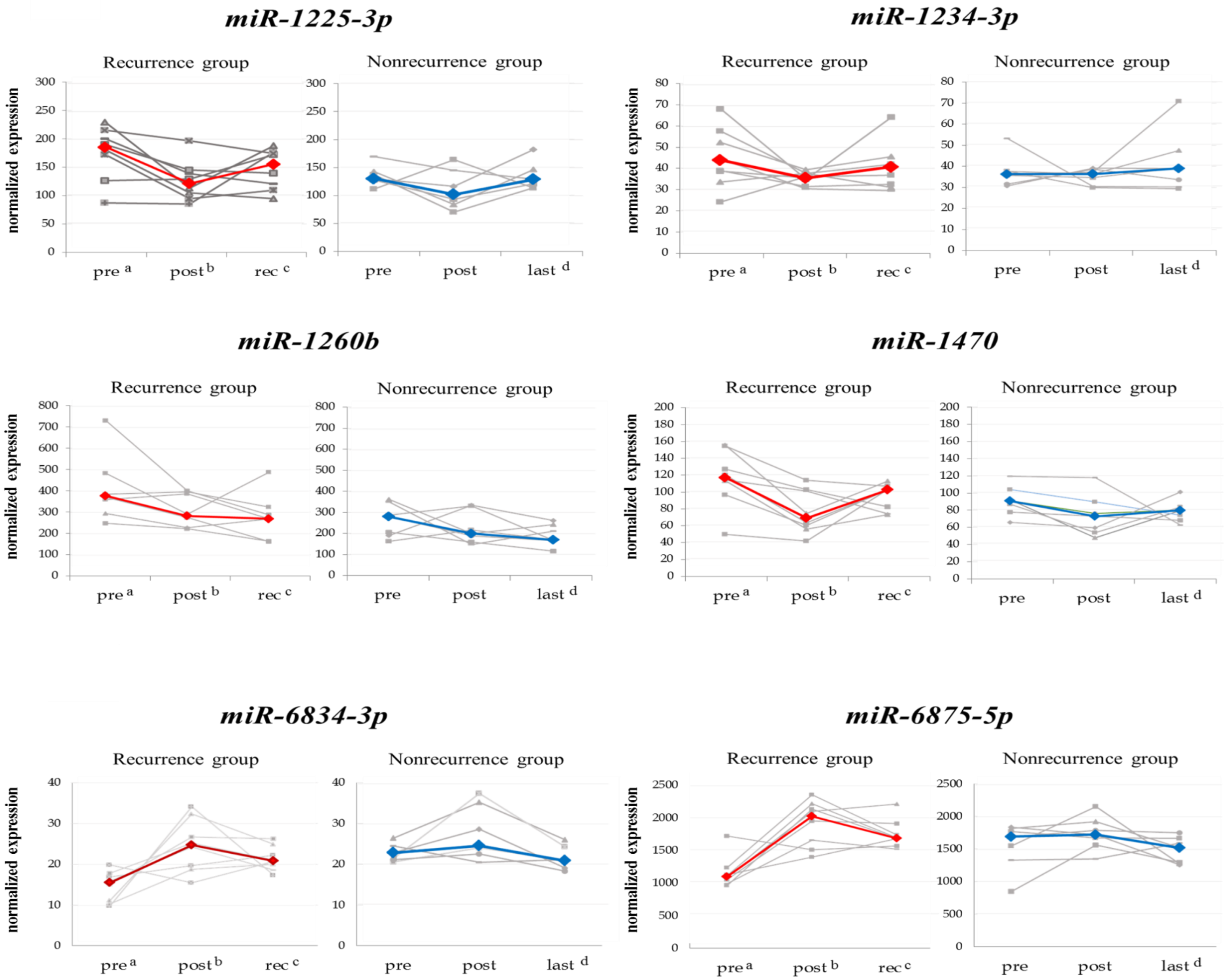

Supplementary Figure S2: Changes in the expression levels of the six candidate miRNAs at the three time points between the recurrence (n = 8) and nonrecurrence (n = 7) groups in patients with bile duct cancer.

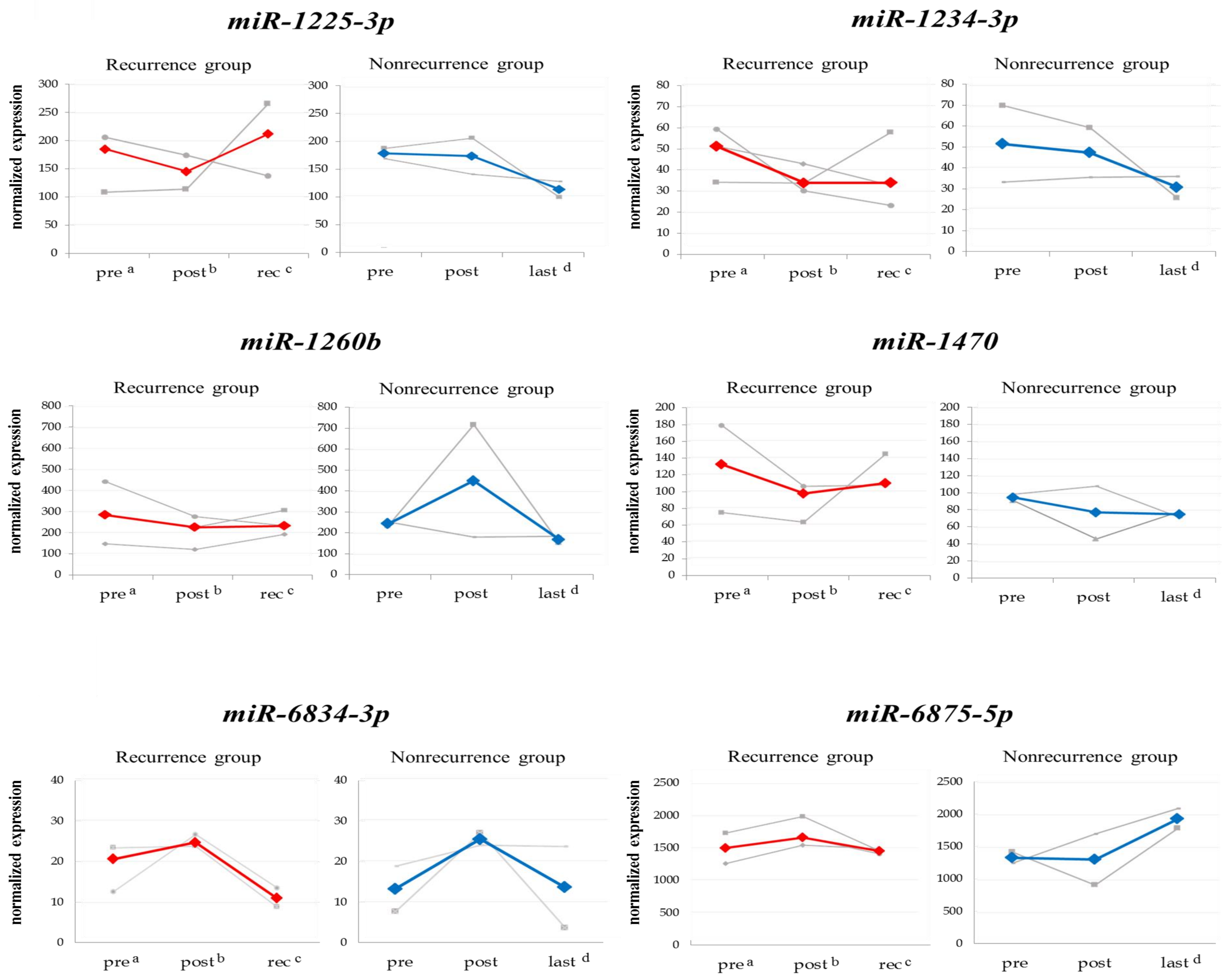

Supplementary Figure S3: Changes in the expression levels of the six candidate miRNAs at the three time points between the recurrence (n = 3) and nonrecurrence (n = 2) groups in patients with gallbladder cancer.

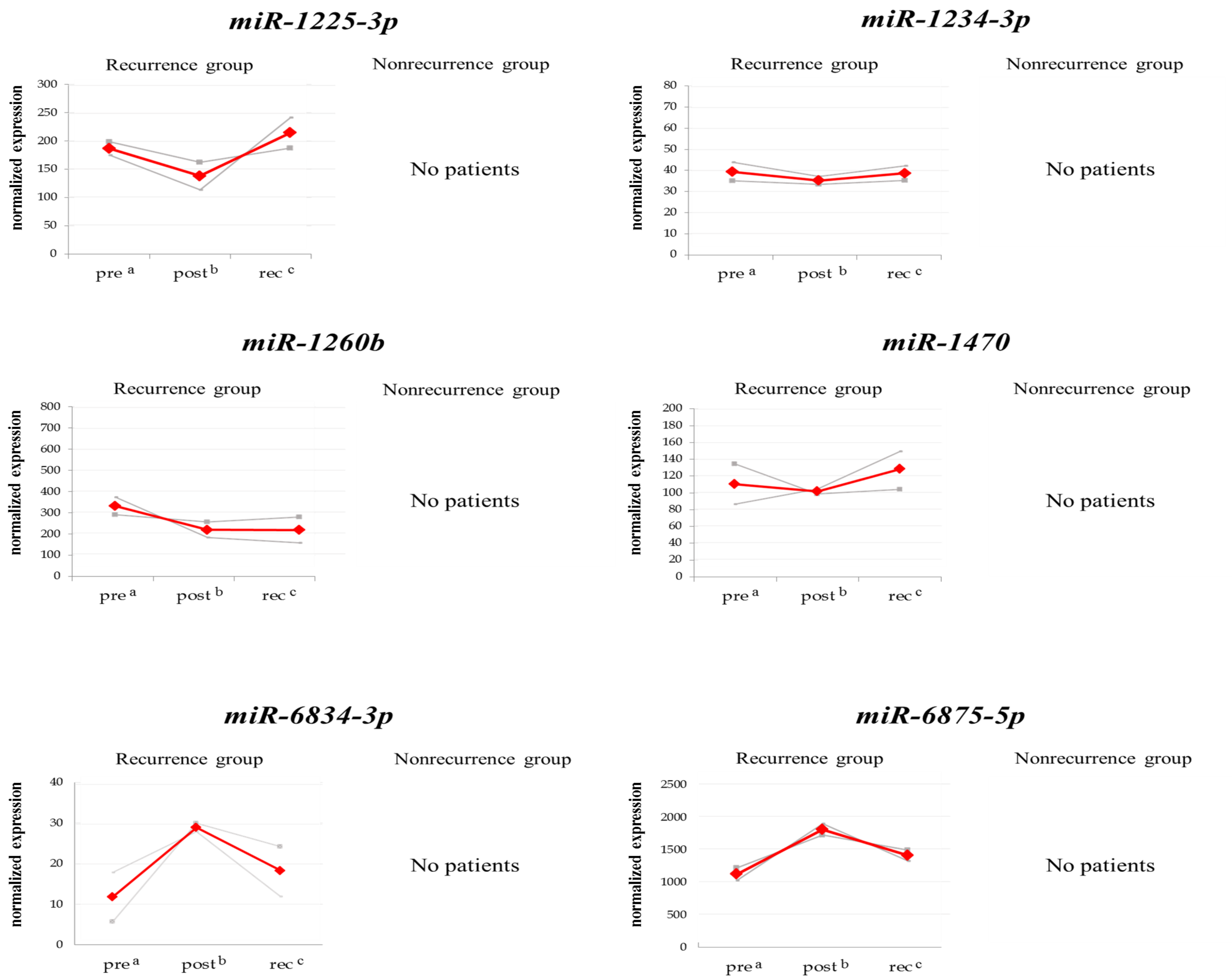

Supplementary Figure S4: Changes in the expression levels of the six candidate miRNAs at the three time points between the recurrence (n = 2) and nonrecurrence (n = 0) groups in patients with ampulla vater cancer.

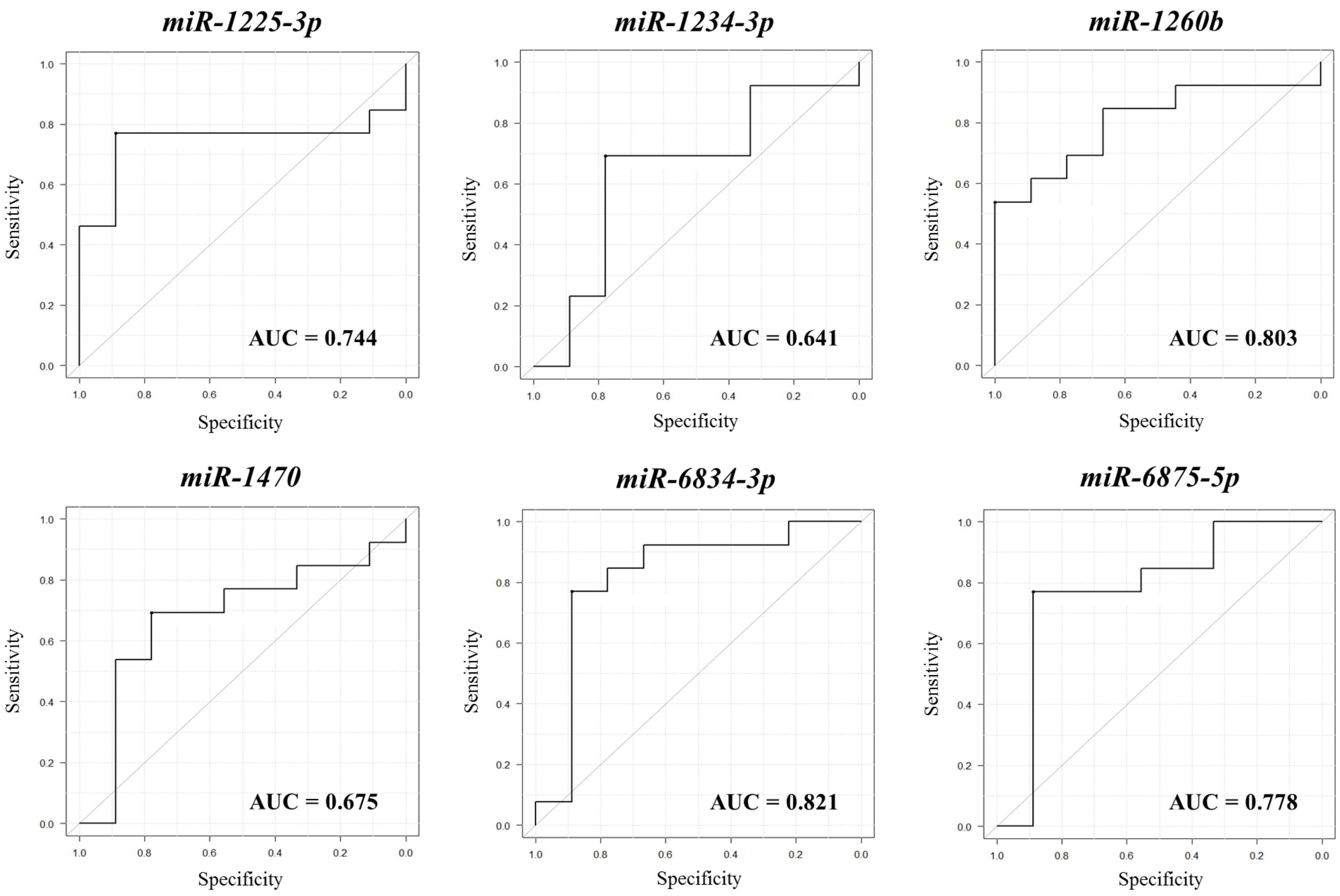

Supplementary Figure S5: Receiver operator characteristic (ROC) curve analysis of the six candidate miRNAs. Area under the curve (AUC) values are shown on the graphs.

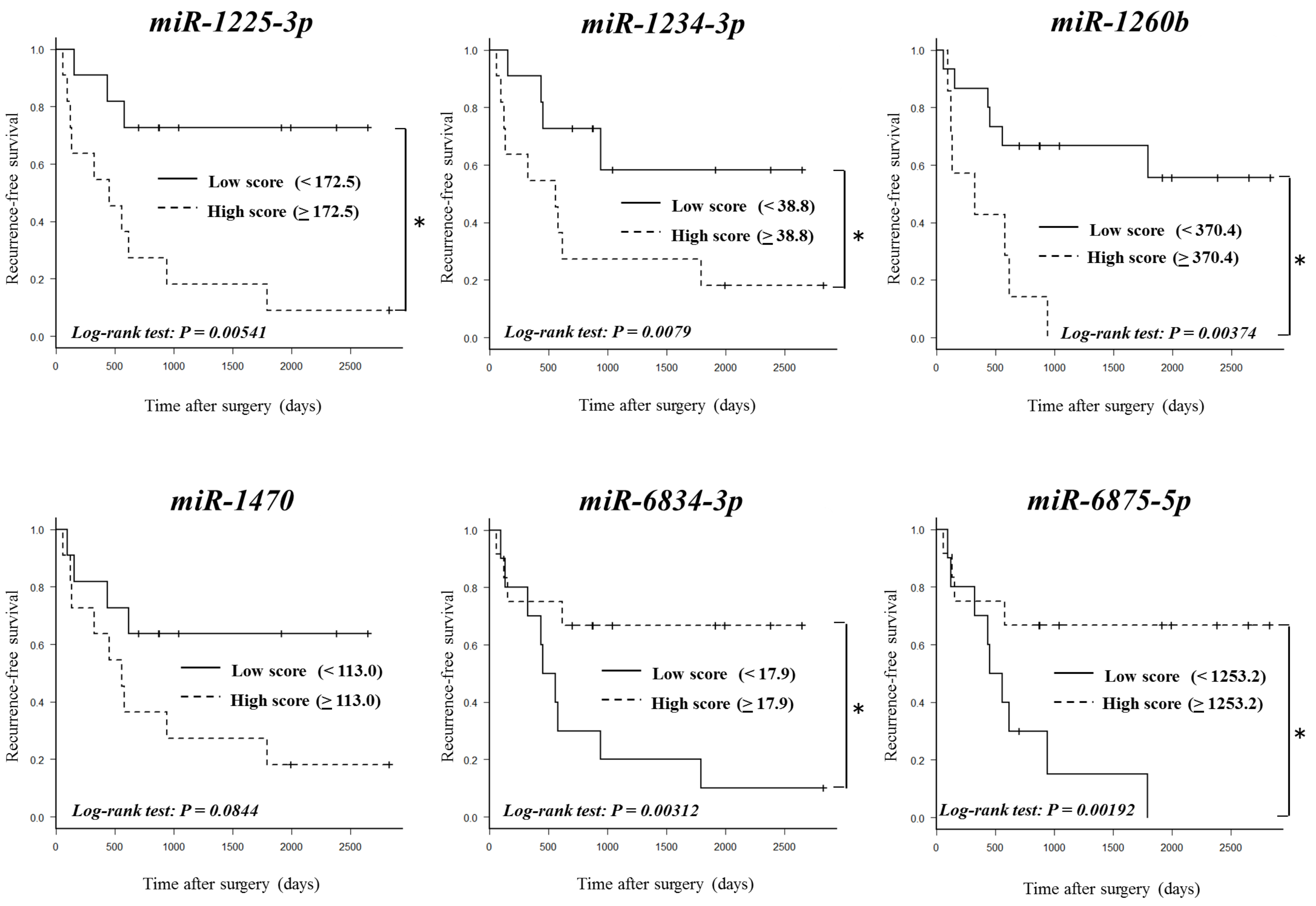

Supplementary Figure S6: Associations of the six candidate miRNAs at the pre-operative time point with recurrence-free survival (RFS) in patients with BTC after radical surgery. Kaplan-Meier graphs showing the probabilities of RFS in the enrolled patients according to the expression levels of the six candidate miRNAs. Log-rank tests were used to analyse the significance of differences. *$P < 0.05$.

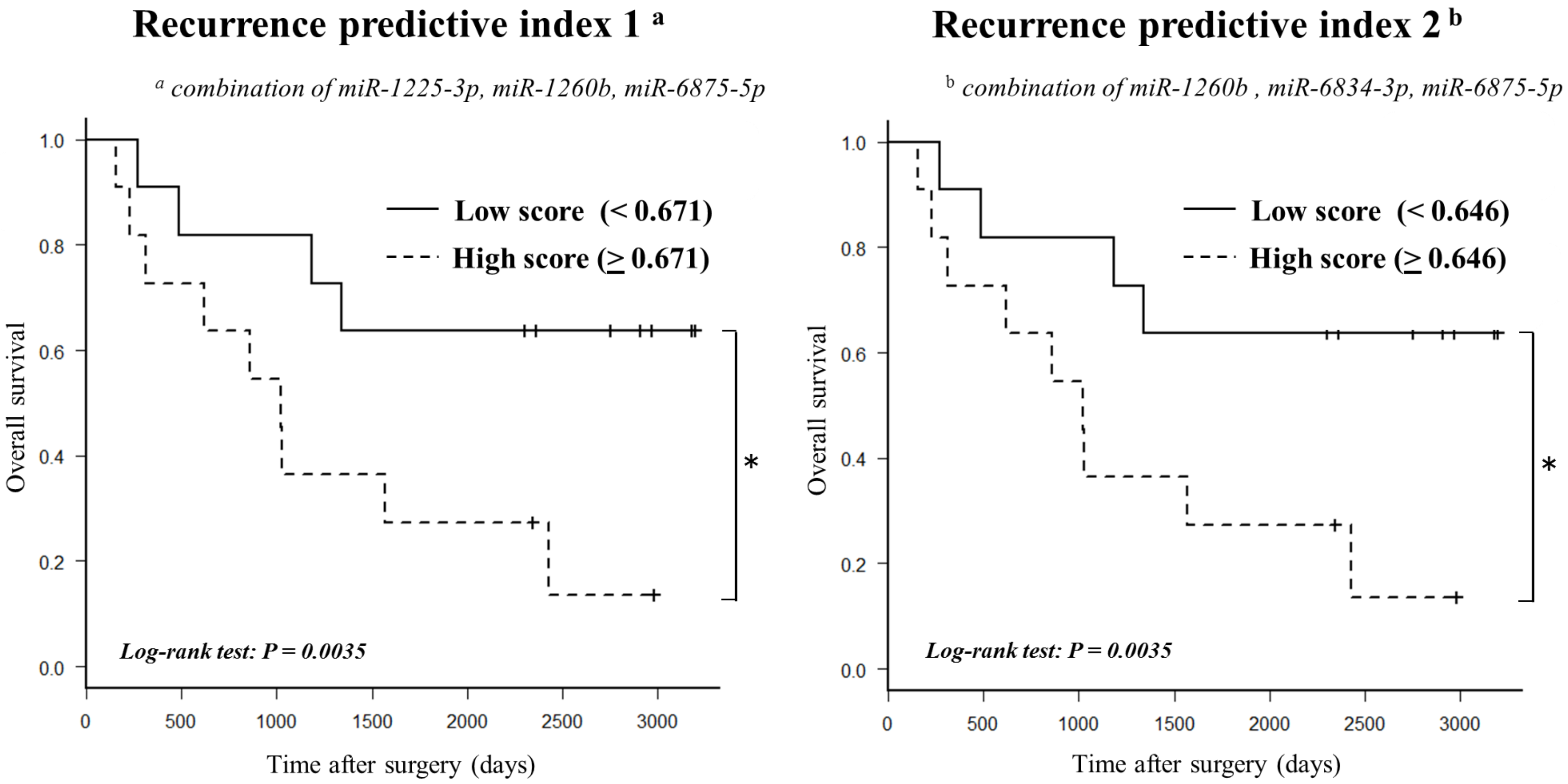

Supplementary Figure S7: Association of recurrence predictive indices using combinations of candidate miRNAs with overall survival (OS) in patients with BTC after radical surgery. Kaplan-Meier graphs representing the probabilities of OS in the enrolled patients according to the recurrence predictive index scores. Log-rank tests were used to analyse the significance of differences. *$P < 0.05$.

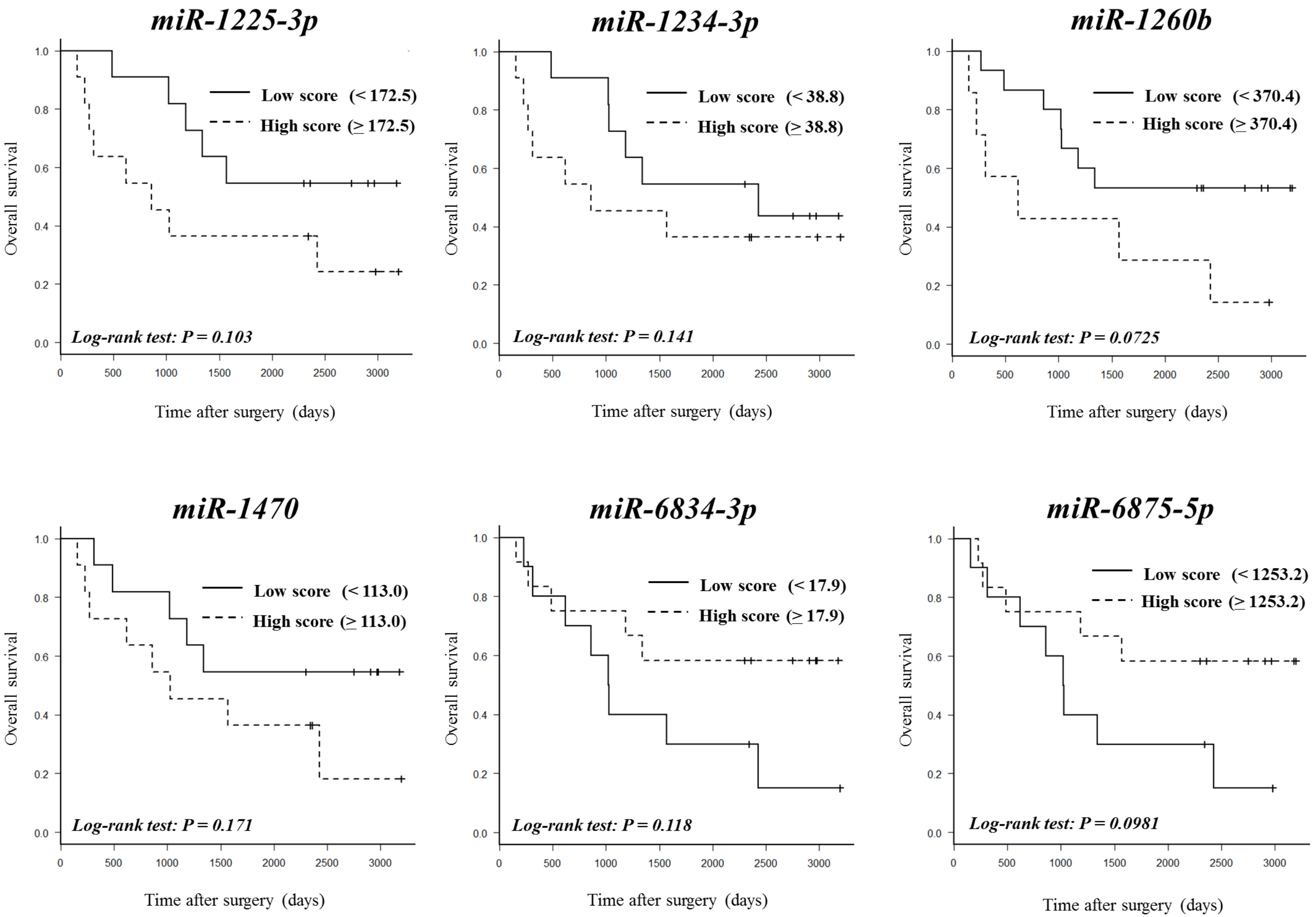

Supplementary Figure S8. Associations of the six candidate miRNAs at the pre-operative time point with overall survival (OS) in patients with BTC after radical surgery. Kaplan-Meier graphs showing the probabilities of OS in the enrolled patients according to the expression levels of the six candidate miRNAs. *P* values were calculated using log-rank tests.

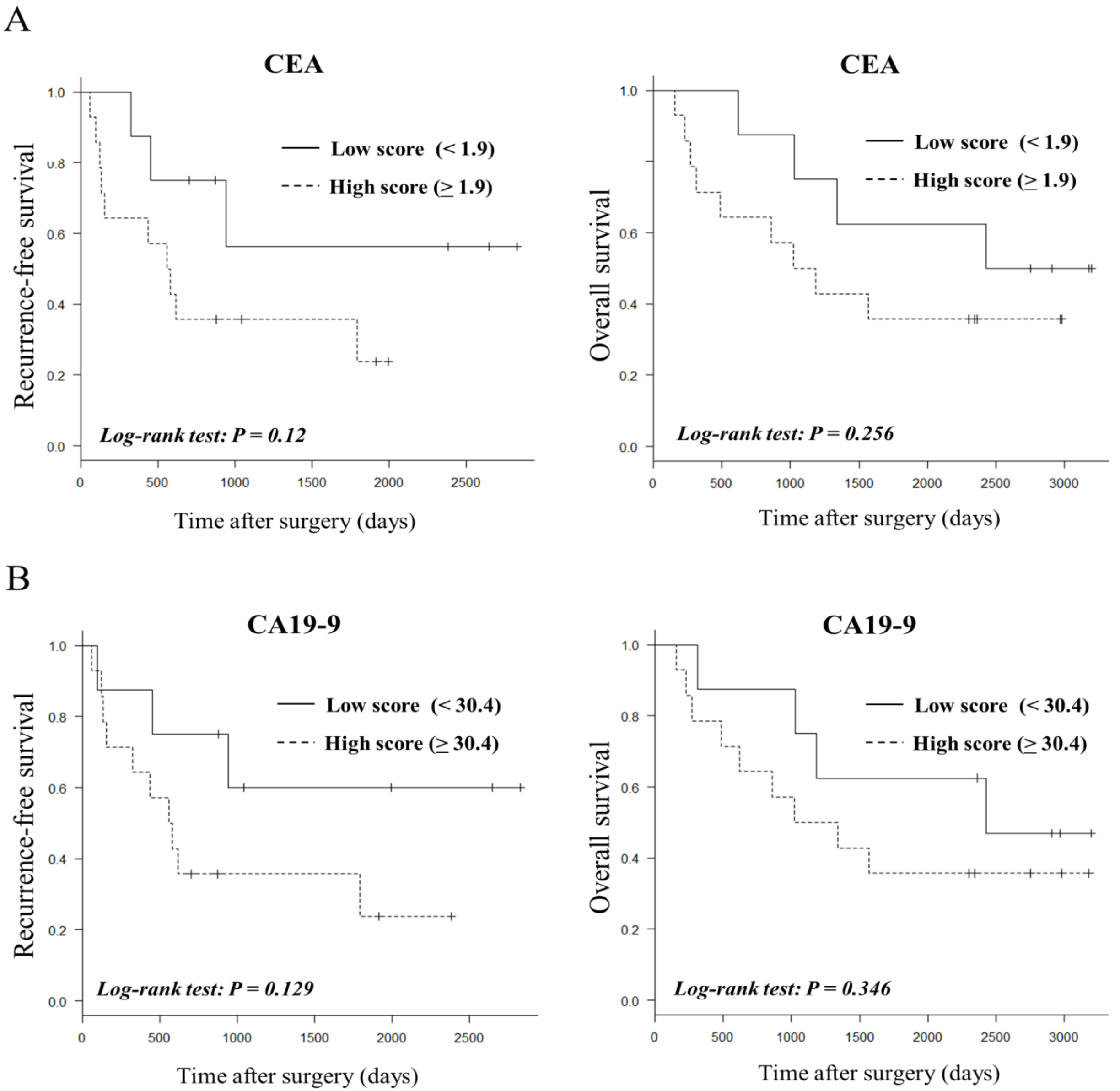

Supplementary Figure S9. Associations of CEA (A) and CA19-9 (B) values at the pre-operative time point with recurrence-free survival (RFS) and overall survival (OS) in patients with BTC after radical surgery. Kaplan-Meier graphs showing the probabilities of RFS and OS in the enrolled patients according to CEA and CA19-9 values. *P* values were calculated using log-rank tests.

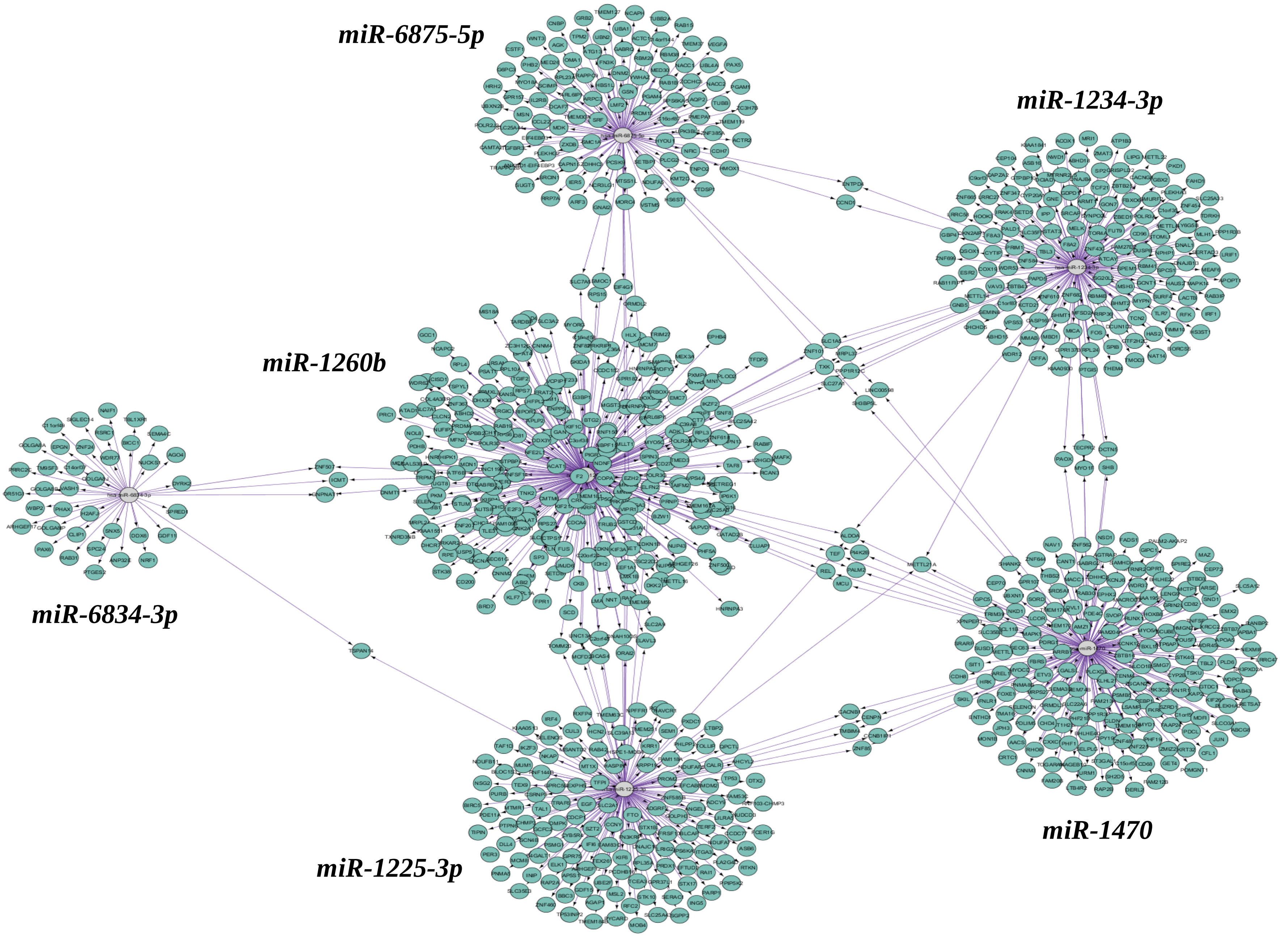

Supplementary Figure S10. Interaction network of six candidate miRNAs and target genes. Gray spots show miRNAs and green spots show target genes.

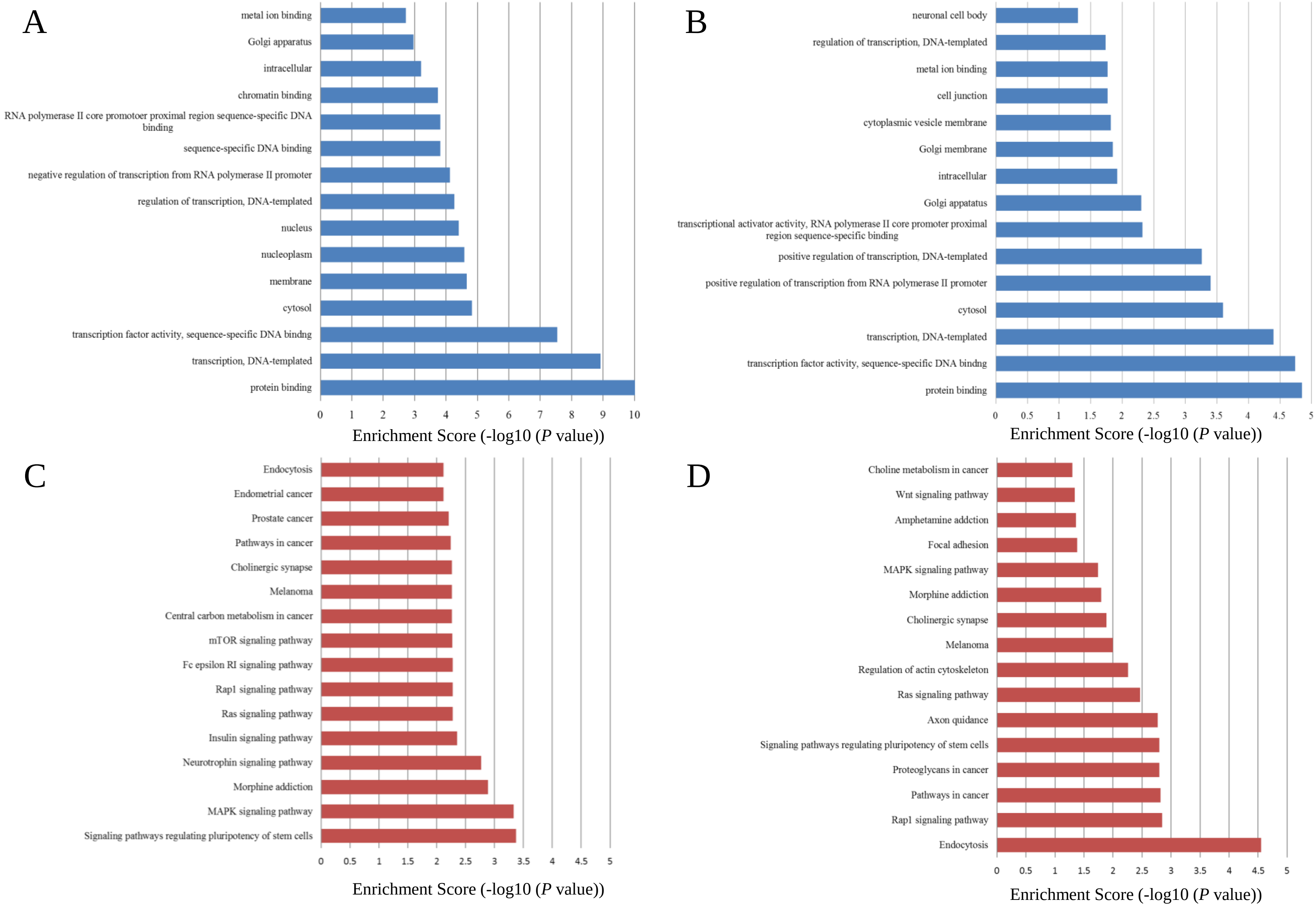

Supplementary Figure S11. The higher enriched GO terms and KEGG pathways by target genes of candidate miRNAs. (A) GO terms of upregulated miRNAs. (B) GO terms of downregulated miRNAs. (C) KEGG pathways of upregulated miRNAs. (D) KEGG pathways of downregulated miRNAs.

Supplementary Table S1: 47 miRNA used as internal control for normalisation.

miR-1227-5p	*miR-1237-5p*	*miR-1268a*	*miR-1268b*	*miR-1469*	*miR-149-3p*	*miR-1908-5p*
miR-1909-3p	*miR-1915-3p*	*miR-3180-3p*	*miR-3196*	*miR-328-5p*	*miR-3621*	*miR-3656*
miR-3663-3p	*miR-3940-5p*	*miR-4281*	*miR-4442*	*miR-4467*	*miR-4492*	*miR-4634*
miR-4651	*miR-4665-5p*	*miR-4674*	*miR-4734*	*miR-4741*	*miR-4763-5p*	*miR-6085*
miR-6087	*miR-6088*	*miR-6125*	*miR-6126*	*miR-663a*	*miR-6724-5p*	*miR-6743-3p*
miR-6749-5p	*miR-6752-5p*	*miR-6765-5p*	*miR-6786-5p*	*miR-6789-5p*	*miR-6791-5p*	*miR-6798-5p*
miR-6803-5p	*miR-6816-5p*	*miR-6850-5p*	*miR-7108-5p*	*miR-8072*		

Supplementary Table S2: Seven (A) and 22 (B) miRNAs in the extraction process of candidate miRNAs related to prediction of recurrence and prognosis as shown in Figure 1.

A) Upregulated miRNAs

No.	miRNA	Median Expression[a]				Difference the expression (pre versus post)[b]	Recurrence-free survival analysis[c]
		Pre (n = 22)	Post (n = 22)	Rec (n = 13)	Last (n = 9)	*P* value	*P* value
**1.**	***miR-1225-3p***	**170.50**	**122.31**	**173.98**	**126.24**	**<0.001**	**0.006**
**2.**	***miR-1234-3p***	**37.96**	**35.62**	**36.63**	**35.50**	**0.020**	**0.046**
3.	*miR-20a-3p*	1.28	0.00	0.00	0.00	0.002	0.057
**4.**	***miR-1260b***	**292.11**	**226.78**	**269.58**	**169.49**	**3.09.E-05**	**0.068**
**5.**	***miR-1470***	**108.13**	**82.07**	**103.73**	**73.43**	**2.159**	**0.082**
6.	*miR-218-1-3p*	0.00	0.00	0.00	0.00	0.047	0.145
7.	*miR-1245a*	0.00	0.00	0.69	0.00	0.008	0.146

B) Downregulated miRNAs

No.	miRNA	Median Expression[a]				Difference the expression (pre versus post)[b]	Recurrence-free survival analysis[c]
		Pre (n = 22)	Post (n = 22)	Rec (n = 13)	Last (n = 9)	*P* value	*P* value
**1.**	***miR-6875-5p***	**1257.15**	**1720.23**	**1558.03**	**1595.81**	**<0.001**	**0.004**
2.	*miR-4449*	132.17	151.92	151.00	155.97	0.030	0.004
3.	*miR-4433b-3p*	280.20	318.50	287.43	293.58	0.027	0.005
4.	*miR-4463*	2067.81	2450.31	2119.95	2106.62	8.39.E-05	0.006
5.	*miR-6749-5p*	934.50	1064.70	976.13	1013.47	0.022	0.007
6.	*miR-874-5p*	47.81	62.18	45.15	45.24	<0.001	0.009
7.	*miR-6832-3p*	9.43	13.92	10.67	10.52	0.004	0.009
8.	*miR-6777-3p*	50.21	56.94	52.42	52.69	<0.001	0.011
9.	*miR-1254*	63.23	90.56	72.54	62.28	4.10.E-05	0.018
**10.**	***miR-6834-3p***	**18.27**	**24.94**	**20.07**	**20.83**	**<0.001**	**0.019**
11.	*miR-939-3p*	26.76	35.29	26.74	28.17	0.007	0.027
12.	*miR-5705*	20.94	32.67	25.40	24.41	<0.001	0.029
13.	*miR-4763-5p*	36.83	42.99	38.00	38.47	0.005	0.030
14.	*miR-491-3p*	45.25	64.93	63.55	58.23	0.001	0.031
15.	*miR-7113-3p*	58.45	72.78	68.28	62.57	0.002	0.032
16.	*miR-30c-1-3p*	49.11	61.25	42.24	47.85	0.014	0.034

17.	*miR-6840-5p*	5.41	10.71	16.46	9.48	0.007	0.035
18.	*miR-5096*	21.59	43.61	9.39	10.38	0.030	0.037
19.	*miR-1915-5p*	43.42	60.32	35.11	41.76	0.014	0.037
20.	*miR-92a-2-5p*	675.00	948.96	554.65	529.59	0.004	0.043
21.	*miR-4450*	94.68	122.62	102.88	95.39	0.011	0.048
22.	*miR-6726-5p*	1031.50	1303.93	800.11	952.22	0.046	0.047

[a] Median miRNA expression at each time point. Pre, Pre-operative time point; Post, Postoperative time point; Rec, Recurrence time point; Last, Last observation time point

[b] Differences in miRNA expression between pre- and postoperative time point were analyzed by paired t test.

[c] Comparison to Recurrence-free survival using Kaplan-Meier method between the high and low value groups divided by median value.

Supplementary Table S3: Univariate and multivariate analyses of clinical factors related to overall survival in patients with BTC after surgery.

	Univariate			Multivariate		
	**HR**	**95% CI**	***P* value**	**HR**	**95% CI**	***P* value**
Recurrence predictive index 1[a]	**3.349**	**1.020–10.99**	**0.046***	**5.051**	**1.251–20.39**	**0.022***
Recurrence predictive index 2[b]	**3.349**	**1.020–10.99**	**0.046***	**5.051**	**1.251–20.39**	**0.022***
Age at diagnosis	1.043	0.986–1.102	0.141			
Sex (men/women)	1.149	0.314–4.206	0.834			
Disease type of BTC	0.978	0.432–2.214	0.958			
Pathological stage at diagnosis	2.159	0.913–5.106	0.079	1.530	0.365–6.419	0.561
Pathological differentiation	1.187	0.480–2.933	0.711			
Lymph metastasis (yes/no)	**3.293**	**1.055–10.28**	**0.040***	3.921	0.699–21.99	0.120
Pre-operative CA19-9 value	2.225	0.681–7.263	0.185	2.560	0.751–8.724	0.133
Pre-operative CEA value	1.976	0.598–6.534	0.264			

[a] Index extracted by the combination of *miR-1225-3p*, *miR-1260b*, and *miR-6875-5p*

[b] Index extracted by the combination of *miR-1260b*, *miR-6834-5p*, and *miR-6875-5p*

*Significant relationship between clinical parameters and overall survival

Abbreviations: BTC, bile tract cancer; HR, hazard ratio; 95% CI, 95% confidence interval; CA19-9, carbohydrate antigen 19-9; CEA, carcinoembryonic antigen.

Supplementary Table S4: Review of the biological pathways of the six candidate miRNAs involved in predicting recurrence and prognosis in BTC.

	Tumor / disease	Potential target	Pathway	Reference
Upregulated miRNAs				
miR-1225-3p	Hepatitis C	GAB3	IFN/JAK/STAT signaling pathway	57
miR-1234-3p	Gastric cancer	β-catenin gene	β-catenin signaling pathway	58
miR-1260b	NSCLC, HCC, RCC, prostate cancer	PTPRK,RGS22, sFRP1, Dkk2, Smad4	Cell migration, invasion, proliferation, apoptosis, Wnt signaling pathway	59,60,61, 62,
miR-1470	ESCC, breast cancer	MMP2, MMP13, MMP14, Bcl-2, c-Jun	Cell cycle, proliferation, Migration, apoptosis, HER2 signaling pathway	63,64
Downregulated miRNAs				
miR-6834-3p	NA	NA	NA	NA
miR-6875-5p	NA	NA	NA	NA

Abbreviations: BTC, bile tract cancer; GAB3, Grb2-associated binding protein 3; NSCLC, non-small cell lung cancer; HCC, hepatocellular carcinoma; RGS22, regulator of G-protein signaling 22; RCC, renal cell carcinoma; ESCC, esophageal squamous cell carcinoma; MMP, matrix metalloproteinase; Bcl-2, B-cell lymphoma 2; NA, not applicable.
